# Asymmetric Michael Addition in Synthesis of β-Substituted GABA Derivatives

**DOI:** 10.3390/molecules27123797

**Published:** 2022-06-13

**Authors:** Jianlin Han, Jorge Escorihuela, Santos Fustero, Aitor Landa, Vadim A. Soloshonok, Alexander Sorochinsky

**Affiliations:** 1 Jiangsu Co-Innovation Center of Efficient Processing and Utilization of Forest Resources, College of Chemical Engineering, Nanjing Forestry University, Nanjing 210037, China; hanjl@njfu.edu.cn; 2 Departamento de Química Orgánica, Universidad de Valencia, 46100 Burjassot, Spain; jorge.escorihuela@uv.es; 3 Department of Organic Chemistry I, Faculty of Chemistry, University of the Basque Country UPV/EHU, Paseo Manuel Lardizábal 3, 20018 San Sebastián, Spain; a.landa@ehu.es (A.L.); vadym.soloshonok@ehu.eus (V.A.S.); 4 IKERBASQUE, Basque Foundation for Science, Alameda Urquijo 36-5, Plaza Bizkaia, 48011 Bilbao, Spain; 5 V.P. Kukhar Institute of Bioorganic Chemistry and Petrochemistry, The National Academy of Sciences of Ukraine, 1 Murmanska Str., 02094 Kyiv, Ukraine

**Keywords:** pharmaceuticals, neurological drugs, γ-aminobutyric-acid derivatives, asymmetric Michael addition, chiral auxiliaries, enantioselective organocatalysis, chiral metal−ligand complexes

## Abstract

γ-Aminobutyric acid (GABA) represents one of the most prolific structural units widely used in the design of modern pharmaceuticals. For example, β-substituted GABA derivatives are found in numerous neurological drugs, such as baclofen, phenibut, tolibut, pregabalin, phenylpiracetam, brivaracetam, and rolipram, to mention just a few. In this review, we critically discuss the literature data reported on the preparation of substituted GABA derivatives using the Michael addition reaction as a key synthetic transformation. Special attention is paid to asymmetric methods featuring synthetically useful stereochemical outcomes and operational simplicity.

## 1. Introduction

**Figure 1:**
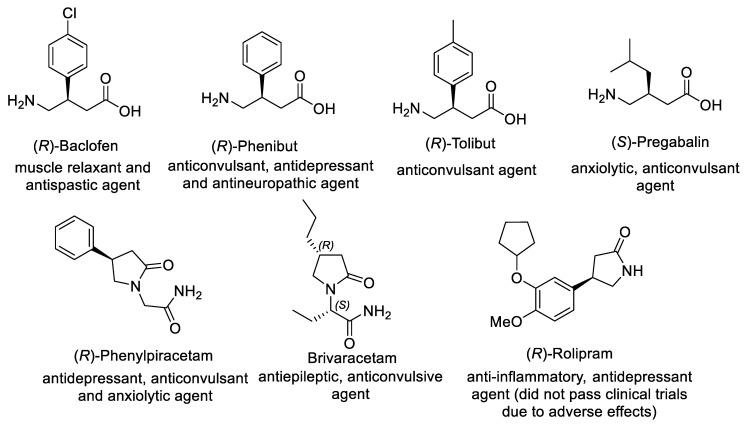
Representative β-substituted GABA derivatives with clinical applications.

Tailor-made amino acids [1] play an indispensable role in the development of modern pharmaceuticals and drug formulations [2,3,4,5,6]. Thus, over 20% of newly approved small-molecule drugs contain structural fragments of AAs [7,8,9]. In particular, γ-aminobutyric-acid (GABA) derivatives bearing β-alkyl or β-aryl substituents, which include baclofen [10], phenibut [11,12,13], tolibut [14], and pregabalin [15,16,17], are recognized as an essential class of marketed pharmaceuticals for the treatment of neurological diseases (Figure 1). Similarly, piracetam-based GABA derivatives such as phenylpiracetam [18], brivaracetam [19,20,21], and rolipram [22] are also developed as pharmaceuticals. Introduction of β-alkyl or β-aryl substituent in the GABA backbone allows for the improvement of the lipophilic character of these compounds.

Significantly, biological activity of β-substituted GABA derivatives depends on their absolute configuration. For example, (*R*)-enantiomers of Baclofen (antispastic agent and muscle relaxant) and Phenibut (tranquilizer and anticonvulsant) are considerably more active than the corresponding (*S*)-enantiomers, while anticonvulsant activity of Pregabalin (anti-epilepsy drug) is primarily related to (*S*)-enantiomer [23]. Consequently, considerable efforts were devoted to developing asymmetric synthesis of β-substituted GABA derivatives, including chemical and biocatalytic resolution, asymmetric reduction, desymmetrization, aldol addition, and nucleophilic substitution [24,25,26]. For the past decade, the asymmetric Michael addition of carbon nucleophile to α,β-unsaturated compound-bearing nitro or carbonyl groups gained impressive progress, providing straightforward access to γ-nitrocarbonyl compounds as key chiral intermediates that can be converted into β-substituted GABA derivatives via subsequent transformation of functional groups. This review focuses on the application of the asymmetric Michael addition performed with the use of chiral auxiliaries as well as in the presence of chiral catalysts, especially chiral metal-free organocatalysts, in synthesis of β-substituted GABA derivatives.

## 2. Michael Addition of Carbonyl Compounds to α,β-Unsaturated Nitroalkenes

**Scheme 1:**
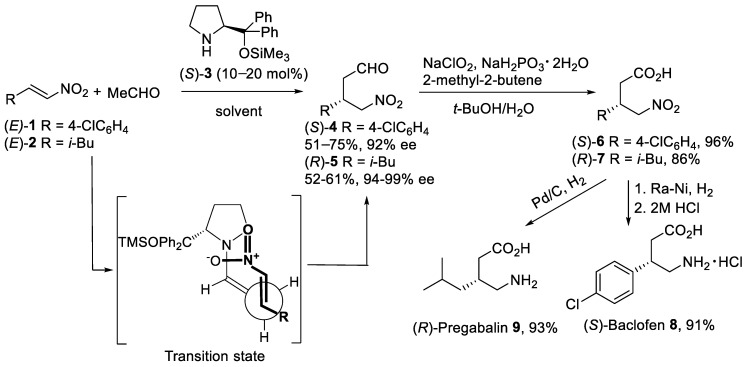
Organocatalytic asymmetric Michael reaction of acetaldehyde with nitroalkenes (*E*)-**1** and (*E*)-**2**.

The proline-catalyzed asymmetric Michael addition reaction [27] of acetaldehyde with α,β-unsaturated nitroalkenes attracted considerable attention as synthetically useful routes to β-substituted derivatives of GABA. Thus, the addition of acetaldehyde to the nitroolefins (*E*)-**1** and (*E*)-**2** (Scheme 1) was carried out in the presence of enantiomerically pure (*S*)-diphenylprolinol silyl ether **3** as the catalyst [28,29]. After optimization of reaction conditions, the asymmetric Michael addition proceeded efficiently with 10–20 mol% of organocatalyst (*S*)-**3** in such solvents as MeCN, DMF/*i*-PrOH, and 1,4-dioxane to afford γ-nitro aldehydes (*S*)-**4** and (*R*)-**5** in reasonable yield and excellent enantiomeric excess. Oxidation of γ-nitro aldehydes (*S*)-**4** and (*R*)-**5** was successfully performed in aqueous *t*-BuOH using NaClO_2_ and NaH_2_PO_4_ with 2-methyl-2-butene as a chlorine scavenger to afford carboxylic acids (*S*)-**6** and (*R*)-**7** in good to excellent yields [30]. The reduction of the nitro acid (*S*)-**6** with Raney Ni in MeOH gave, after treatment with aqueous HCl, (*S*)-Baclofen **8** as hydrochloride salt in 91% yield. (*R*)-Pregabalin **9** was also synthesized by the reduction of the nitro group in (*R*)-**7** under Pd/C in 93% yield. The mechanism of the asymmetric Michael addition reaction of acetaldehyde with nitroalkenes promoted by diphenylprolinol silyl ether involves the formation of the enamine as a nucleophile. Thus, the organocatalyst would react with the acetaldehyde forming *anti*-enamine with the double bond oriented away from the diphenylsiloxymethyl group. In this case, the (diphenylmethyl)trimethylsiloxy group provides the formation of *anti*-enamine and shielding one face of the enamine double bond. The *anti*-enamine would add stereoselectively to the nitroolefin via the acyclic synclinal transition state proposed by Seebach [31] as shown in Scheme 1.

**Scheme 2:**
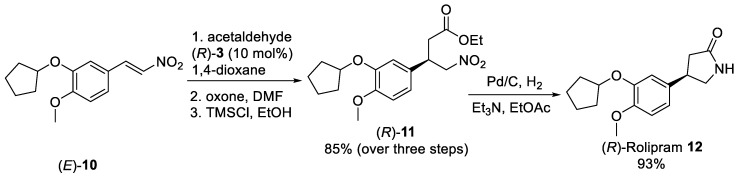
Synthetic approach of (*R*)-Rolipram **12** employing the organocatalyzed asymmetric Michael addition acetaldehyde to nitro olefin (*E*)-**10**.

In a similar way, the nitro olefin (*E*)-**10** (Scheme 2), easily available from isovanillin via *O*-alkylation and Henry condensation, reacted with acetaldehyde in the presence of a catalytic amount of diphenylprolinol silyl ether (*R*)-**3** (10 mol%) affording corresponding nitro aldehyde adduct which upon oxidation with oxone and esterification successfully transformed into ester derivative (*R*)-**11** in 85% yield [32]. The nitroester (*R*)-**11** underwent intramolecular reductive lactamization under H_2_ in presence of catalytic amount of Pd/C to furnish (*R*)-Rolipram **12** in 93% yield and >99% ee. The present method was also utilized to prepare enantiomerically pure (*S*)-Rolipram using (*S*)-diphenylprolinol silyl ether-mediated asymmetric Michael addition reaction as the key step. Recently, water-soluble diarylprolinol silyl ether containing the dimethylamine functionality was found to be very effective for the Michael additions of the acetaldehyde with nitroolefins. These reactions took place in brine with good yields and high enantioselectivities for a broad range of nitroolefins [33,34,35].

**Scheme 3:**
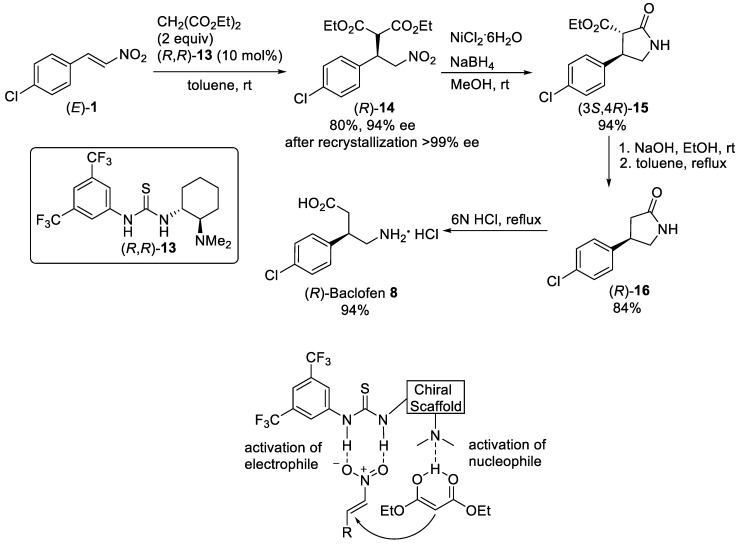
Enantioselective Michael reaction of (*E*)-**1** with diethyl malonate in the presence of thiourea (*R*,*R*)-**13**.

Enantioselective conjugate addition of malonates and their equivalents to nitroolefins promoted by bifunctional organocatalysts bearing a hydrogen-bonding donor group and Lewis base (tertiary amine) is considered as one of the most simple and efficient routes for constructing chiral β-substituted derivatives of GABA and their lactam analogs, especially from the perspective of green chemistry. The success of bifunctional organocatalysts was based on their ability to increase the reactivity of both nitroolefins and nucleophiles as well as control the approach of nucleophiles to nitroolefins in the transition state [36,37]. For example, the Michael reaction of nitroalkene (*E*)-**1** (Scheme 3) with diethyl malonate was performed in the presence of Takemoto thiourea catalyst (*R*,*R*)-**13** bearing 3,5-bis(trifluoromethyl)benzene and tertiary amino group [38]. The use of 2 equiv of diethyl malonate in toluene and 10 mol% catalyst loading was required to provide the Michael adduct (*R*)-**14** in 80% yield with 94% ee. A single recrystallization made it possible to increase the enantiomeric purity of the product (*R*)-**14** to 99% ee. Then, reductive cyclization of (*R*)-**14** with NaBH_4_/NiCl_2_ in methanol gave γ-lactam (3*S*,4*R*)-**15** as thermodynamically more stable *anti*-diastereomer [39,40], which after hydrolysis and decarboxylation was converted into lactam (*R*)-**16** in 84%. Finally, acidic hydrolysis of (*R*)-**16** gave (*R*)-Baclofen **8** as hydrochloride salt in 94% yield. A high level of enantioselectivity in organocatalytic Michael addition reaction was achieved as a result of dual activation through deprotonation of the acidic proton of diethylmalonate by tertiary amino group of the organocatalyst and the hydrogen-bond formation between the nitro group and the thiourea moiety [41].

**Scheme 4:**
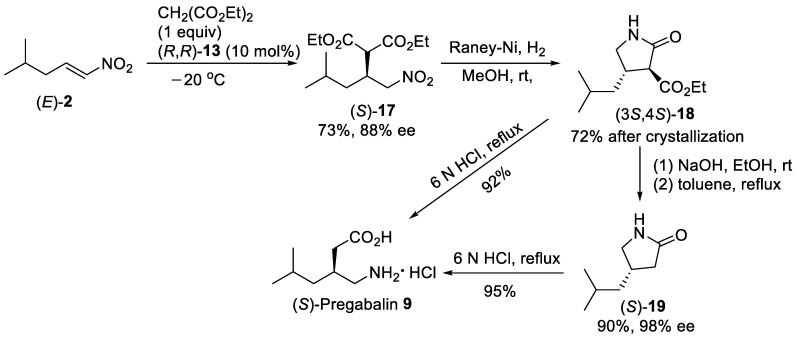
Enantioselective Michael reaction of (*E*)-**2** with diethyl malonate in the presence of thiourea (*R*,*R*)-**13** under solvent-free conditions.

Under solvent-free conditions reducing the amount of diethyl malonate from 2 to 1 equiv did not significantly influence both the yield and enantioselectivity of Michael addition to nitroalkenes promoted by thiourea (*R*,*R*)-**13** catalysts. Thus, Michael addition of diethyl malonate to alkyl-substituted nitroalkene (*E*)-**2** (Scheme 4) in the presence of 10 mol% of (*R*,*R*)-**13** under solvent-free conditions produced the nitro ester (*S*)-**17** in 73% yield and 88% enantiomeric excess [42]. Hydrogenation of (*S*)-**17** over Raney Ni provided the pyrrolidin-2-one (3*S*,4*S*)-**18** [43] in 72% after crystallization, which was subjected to ester hydrolysis followed by decarboxylation to give γ-lactam (*S*)-**19** in 90% yield and 98% enantiomeric excess. Hydrolysis of γ-lactam (*S*)-**19** with 6N HCl gave the enantiomerically pure (*S*)-Pregabalin **9** hydrochloride in 95% yield. Additionally, treatment of pyrrolidin-2-one derivative (3*S*,4*S*)-**18** with 6N HCl at reflux also directly produced the (*S*)-Pregabalin **9** hydrochloride in 92% yield.

**Scheme 5:**
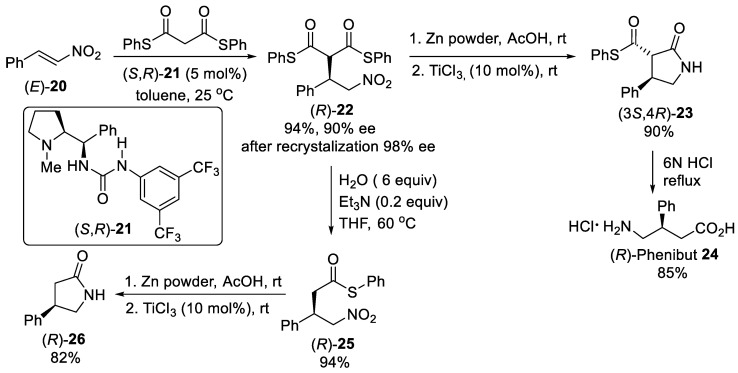
Enantioselective Michael addition of diphenyl dithiomalonates to β-nitrostyrene (*E*)-**20** catalyzed by L-proline-derived urea (*S*,*R*)-**21**.

A series of Takamoto-type bifunctional organocatalysts were examined for the asymmetric Michael addition of malonate derivatives to nitroalkenes and showed very effective catalytic activity. For example, L-proline-derived bifunctional urea-pyrrolidine organocatalyst (*S*,*R*)-**21** (Scheme 5) was demonstrated to catalyze the enantioselective Michael addition of diphenyl dithiomalonates to nitrostyrene (*E*)-**20** in toluene as the best solvent at 25 °C [44]. The reaction performing with 5 mol% of (*S*,*R*)-**21** was complete in 1.5 h, affording Michael adduct (*R*)-**22** in 90% enantiomeric excess, which was improved to 98% after recrystallization from ethanol. Reduction of the nitro group using zinc in acetic acid and substoichiometric amounts of TiCl_3_ followed by intermolecular cyclization enabled the formation of the lactam (*R*)-**23** in excellent yields of 90%. Hydrolysis of the lactam (*R*)-**23** was finally achieved with 6N HCl and the resulting (*R*)-Phenibut **24** was isolated as hydrochloride in 85% yield. Additionally, mild basic hydrolysis-decarboxylation of Michael adduct (*R*)-**22** provided γ-nitrothioester (*R*)-**25** in 94% yield, which reduced and cyclized under the above described conditions (Zn/AcOH/TiCl_3_) to lactam (*R*)-**26** that was isolated in 82% yield.

**Scheme 6:**
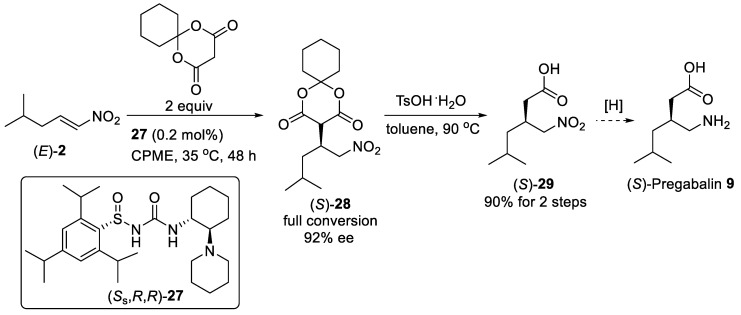
Catalytic enantioselective addition of cyclohexyl Meldrum’s acid to nitroalkene (*E*)-**2** using *N*-sulfinyl urea catalysis (*S*_s_,*R*,*R*)-**27**.

When the addition of cyclohexyl Meldrum’s acid to aliphatic nitroalkene (*E*)-**2** (Scheme 6) was carried out with only 0.2 mol% of *N*-sulfinyl urea catalysis (*S*_s_,*R*,*R*)-**27** in cyclopentyl methyl ether (CPME), complete conversion of starting nitroalkene (*E*)-**2** proceeded at 35 °C for 48 h providing the Michael adduct (*S*)-**28** in 92% ee [45]. Using *n*-sulfinyl urea catalysis (*S*_s_,*R*,*R*)-**27** bearing cyclic tertiary amine was found to be essential for achieving high conversion and enantioselectivity. Direct hydrolysis/decarboxylation of addition product (*S*)-**28** without purification led to one mole scale synthesis of γ-nitroacid (*S*)-**29** in 90% overall yield from nitroalkene (*E*)-**2**. (*S*)-Pregabalin **9** could be provided by heterogeneous catalytic hydrogenation of (*S*)-**29**.

**Scheme 7:**
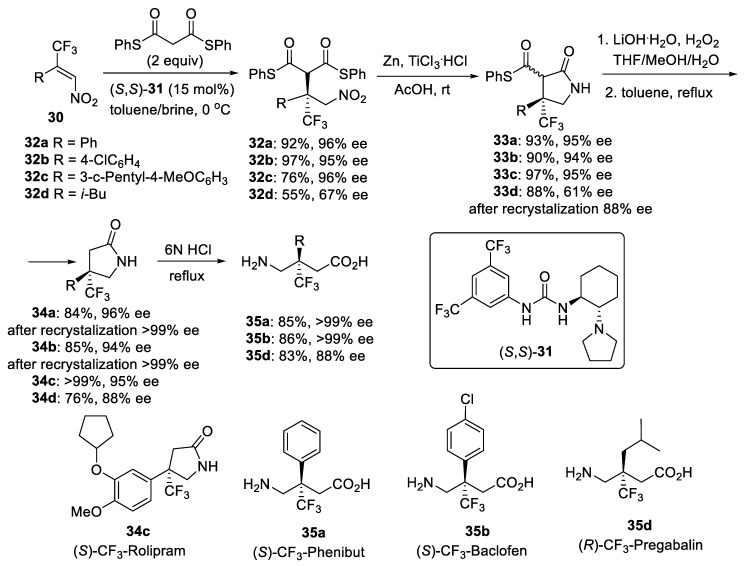
Synthesis of GABA derivatives with a trifluoromethylated quaternary stereogenic center.

Another urea catalyst (*S*,*S*)-**31** (Scheme 7) containing the pyrrolidine moiety was designed to catalyze in brine media [46] the enantioselective addition of dithiomalonate to otherwise unreactive β-CF_3_-β,β-disubstituted nitroalkenes **30** providing corresponding Michael adducts **32** with a β-trifluoromethylated quaternary stereocenter [47]. In brine media the use of 15 mol% of urea catalyst (*S*,*S*)-**31** as well as the addition of *o*-xylene as additive was required for obtaining high yield and enantioselectivity of adducts **32** at 0 °C. In the absence of cosolvent, the achieved enantioselectivity of Michael adducts **32** was lower. These results could be explained by enforced hydrophobic interactions between catalysts and substrates when the reaction was carried out in brine media. Under optimal conditions, trifluoromethylated nitroalkenes **30** having substituted phenyl and *iso*-butyl groups were converted into the corresponding products **32** in 55–97% yields with 67–96% ee. So-obtained Michael products **32** were further subjected to reductive cyclization to furnish γ-lactam thioesters **33**. Hydrolysis of γ-lactam thioesters **33** followed by a decarboxylation provided β-trifluoromethylated analogues of rolipram **34c**, phenibut **35a**, baclofen **35b**, and pregabalin **35d**. The enantiopurity of γ-lactam thioester **33d** and γ-lactams **34a**,**b** could be improved by recrystallization.

**Scheme 8:**
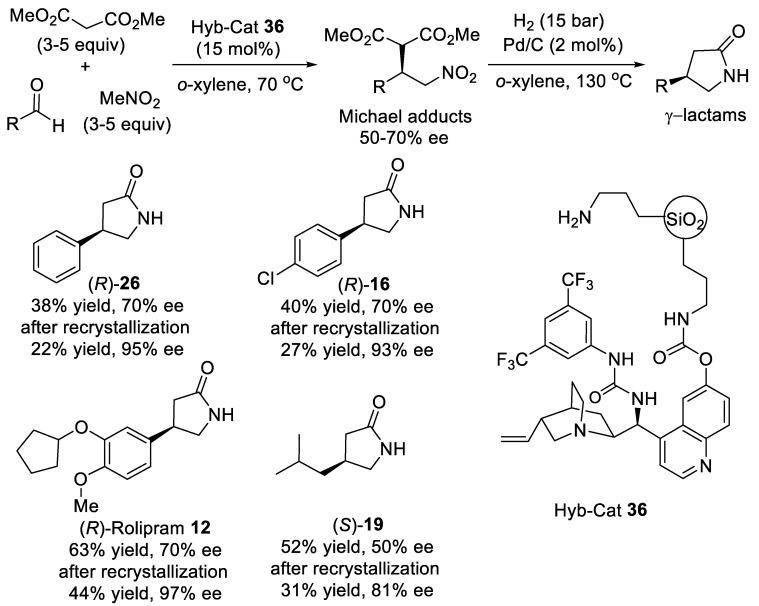
Asymmetric three-component reaction of aldehydes, nitromethane, and malonate.

A three-component reaction of aromatic or aliphatic aldehydes, nitromethane and dimethyl malonate was found to catalyze by mesoporous siliceous material **36** (Scheme 8) incorporating urea-modified quinidine and propylamine groups in *o*-xylene at 70 °C leading to corresponding Michael adducts in reasonable yield [48,49]. The organic–inorganic hybrid catalyst **36** enabled access to Michael adducts with modest-to-good enantiomeric excess (50–70%). After removing the excess nitromethane by distillation, the *o*-xylene solution of Michael adducts was subjected to heterogeneous catalytic hydrogenation of the nitro group followed by cyclization and thermal decarboxylation to give γ-lactams with retention of enantiopurity. Thus, after column chromatography and recrystallization, (*R*)-Rolipram **12** and precursors of Phenibut (*R*)-**26**, Baclofen (*R*)-**16** and Pregabalin (*S*)-**19** were obtained in two-pot multicomponent operations with enantiopurity ranging from 81% ee to 97% ee. The solid hybrid catalyst could be reused three times without loss of activity.

**Scheme 9:**
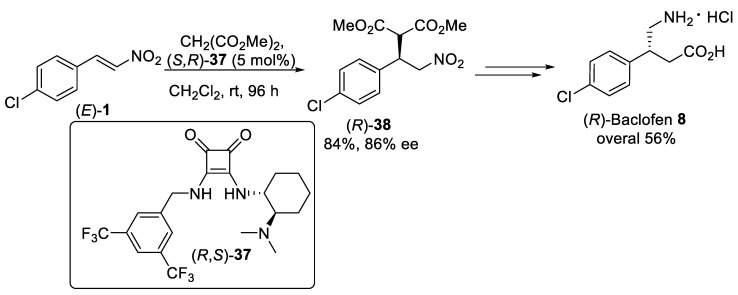
Michael addition of dimethyl malonate to β-nitrostyrene (*E*)-**1** using squaramide organocatalysis (*R*,*S*)-**37**.

Conformationally rigid cyclobutene-ring-derivative squaramide (*R*,*S*)-**37** (Scheme 9) was shown to function in a complementary manner to thioureas as an effective hydrogen-bonding bifunctional organocatalyst for the Michael addition of dimethyl malonate to aromatic nitroalkene (*E*)-**1** [50,51]. It was supposed that two H-bonds formed between the squaramide (*R*,*S*)-**37** organocatalyst, incorporating tertiary nitrogen as Lewis base and chiral scaffold, and the nitroalkene substrate. Performing the reaction in dichloromethane as solvent with 5 mol% catalyst loading afforded the Michael product (*R*)-**38** in 84% yield and 86% ee, albeit in a long reaction time. Polymer-immobilized squaramide was also effective for the Michael addition of dimethyl malonate to nitroalkene (*E*)-**1**, but significant loss of activity was observed in the second and third cycles. The adduct (*R*)-**38** was further transformed to (*R*)-baclofen **8** hydrochloride in 56% overall yield through the procedure exemplified in Scheme 1.

**Scheme 10:**
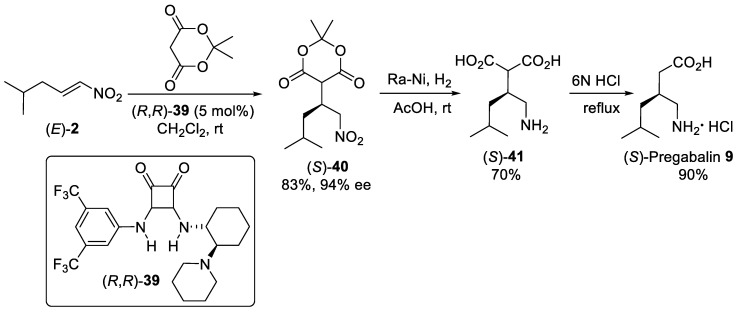
The Michael addition of Meldrum’s acid to nitroalkene (*E*)-**2** catalyzed by squaramide (*R*,*R*)-**39** catalyst.

Squaramide (*R*,*R*)-**39** (Scheme 10), which possess an piperidine unit, was useful for the activation of Meldrum’s acid in enantioselective Michael addition to aliphatic nitroalkenes. The reaction of Meldrum’s acid and nitroalkene (*E*)-**2** was carried out with catalyst loading of 5 mol% in dichloromethane as the best solvent to obtain the nitro derivative (*S*)-**40** in 83% yield and 94% enantiomeric excess [52]. Squaramide (*R*,*R*)-**39** showed higher catalytic activity and enantioselectivity compared to quinidine-based thioureas organocatalysts [53]. Single-step catalytic hydrogenation and acid-catalyzed deprotection of (*S*)-**40** over Raney Ni in acetic acid than decarboxylation of the resulting malonic-acid derivative (*S*)-**41** with 6*N* HCl afforded (*S*)-Pregabalin **9** hydrochloride in 52% yield for three steps.

**Scheme 11:**
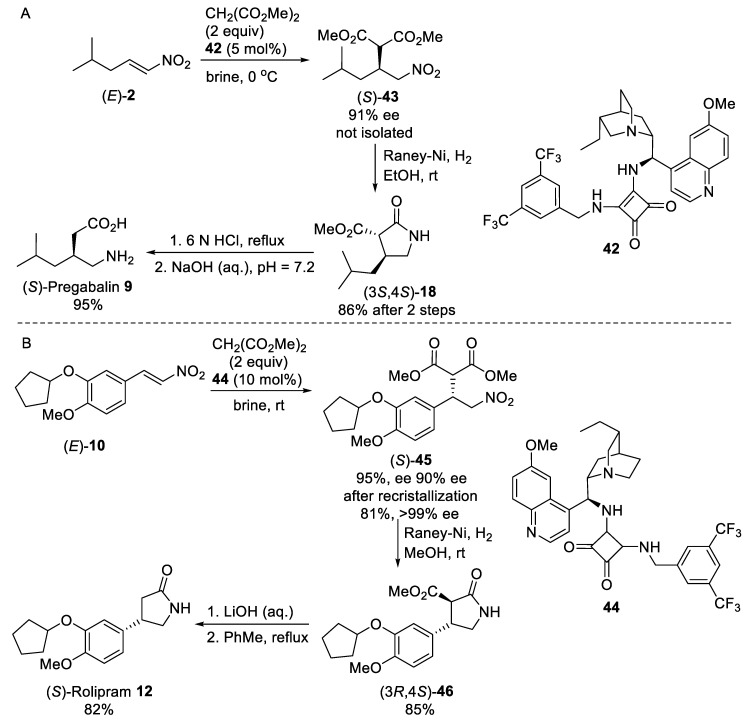
Enantioselective syntheses of (*S*)-Pregabalin **9** (**A**) and (*S*)-Rolipram **12** (**B**) under “on water” conditions.

The use of catalyst **42** (Scheme 11A) incorporating squaramide and hydroquinidine units allowed one to perform an enantioselective Michael addition of malonate to nitroolefin (*E*)-**2** in brine due to the “hydrophobic hydration effect” [54,55]. Under these conditions, hydrophobic hydroquinidine-squaramide catalyst **42** directed the reaction towards the corresponding Michael adduct (*S*)-**43** with high yield and enantioselectivity [43]. After the extraction of the Michael adduct (*S*)-**43** with methylcyclohexane, catalyst **42** was recoveried in quantitative yield (>99%) by simple filtration permitted the catalyst recycling. Hydrogenation of the crude Michael adduct (*S*)-**43** using Raney Ni followed by hydrolysis of resulting γ-lactam (3*S*,4*R*)-**18** with 6N HCl afforded (*S*)-pregabalin **9** in enantiomerically pure form after simple recrystallization from 2-propanol/water. The developed procedure in brine media was also applied for the scalable synthesis of enantiomerically pure (*S*)-Rolipram **12** (Scheme 11B) from aryl nitroolefin (*E*)-**10** using 10 mol% of hydroquinine-based squaramide catalyst **44**.

**Scheme 12:**
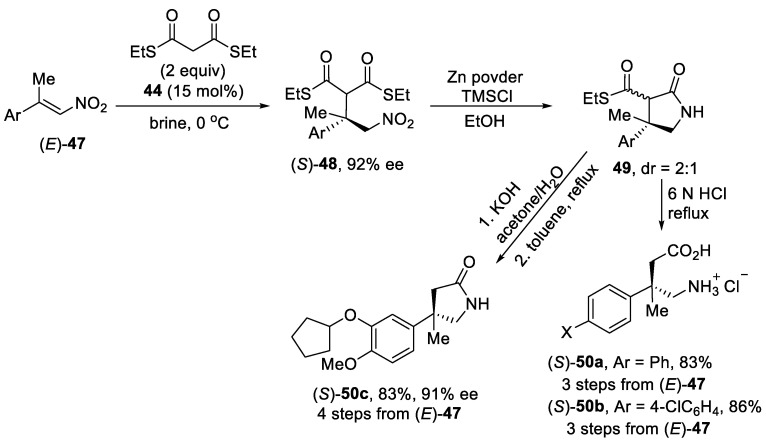
Enantioselective Michael addition reaction of β,β-disubstituted nitroalkenes (*E*)-**47** with dithiomalonate using dihydroquinine-squaramide catalyst.

The hydrophobic dihydroquinine-squaramide derivative **44** (Scheme 11B) also demonstrated its efficiency in brine media for enantioselective Michael addition of the diethyl dithiomalonates to (*E*)-α-methyl-β-nitrostyrenes **47** (Scheme 12) [56]. In these cases, a high loading of **44** (15 mol%) and lowering the reaction temperature to 0 °C were required for the reaction to proceed with high enantioselectivities. At the same time, no reaction was observed when catalyst **44** was used in organic solvents. The resulting adducts (*S*)-**48** could further be converted into GABA analogs bearing an all-carbon quaternary stereocenter at the β-position. After the separation of the catalyst, the crude products (*S*)-**48** were subjected to reduction affording γ-lactam thioesters **49**, which were hydrolyzed with 6N HCl into β-methylated analogs of Phenibut (*S*)-**50a** and Baclofen (*S*)-**50b** as hydrochloride salts. At the same time, the β-methylated analog of Rolipram (*S*)-**50c** was prepared by basic hydrolysis and decarboxylation of corresponding γ-lactam thioester **49**.

**Scheme 13:**
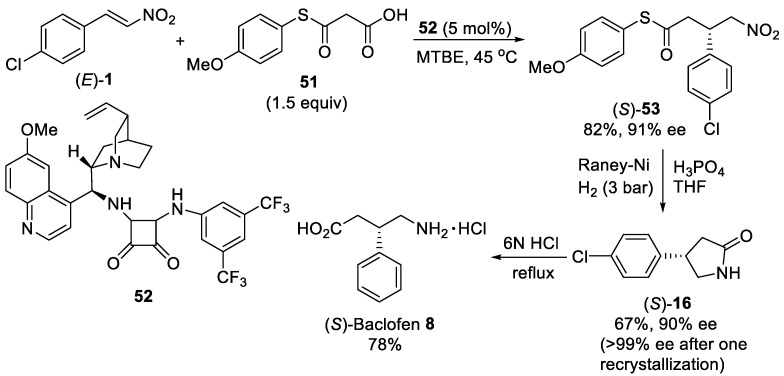
Enantioselective Michael addition of malonic-acid half-thioester **51** to β-nitrostyrene (*E*)-**1**. Synthesis of (*S*)-baclofen **8**.

An enantioselective decarboxylative Michael addition between malonic-acid half-thioester [57] and β-nitroolefins providing access to γ-nitro thioester was demonstrated to catalyze with cinchona-based squaramide [58] and cinchona-based urea [59] organocatalysts under mild reaction conditions. Performing the reaction of malonic-acid half-thioester **51** (Scheme 13) and β-nitrostyrene (*E*)-**1** with loading of 5 mol% of bifunctional quinine-based squaramide **52** as the most active and selective organocatalyst in methyl *tert*-butyl ether (MTBE) at 45 °C resulted in addition product (*S*)-**53** in high yield and excellent enantioselecitivity within 22 h [58]. It is noteworthy that using *E* or *Z* isomers of the starting β-nitrostyrene **1** afforded the (*S*)-enantiomer of the Michael adduct **53** with the same ee value. The nitro thioester (*S*)-**53** was converted with Raney Ni and H_3_PO_4_ in THF by intramolecular cyclization and recrystallization into enantiomerically pure γ-butyrolactam (*S*)-**16**. Finally, hydrolysis of resulting γ-butyrolactam (*S*)-**16** could be performed with 6N HCl under reflux to deliver the (*S*)-Baclofen **8** as its hydrochloride salt in 78% yield.

**Scheme 14:**
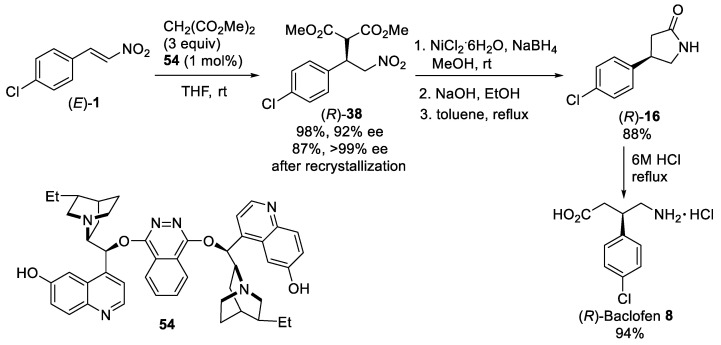
Asymmetric conjugate addition of dimethyl malonate to β-nitrostyrene (*E*)-**1**. Synthesis of (*R*)-Baclofen **8** hydrochloride.

The bifunctional bisalkaloid organocatalyst **54** (Scheme 14) was developed and successfully tested for the enantioselective conjugate addition of malonates to nitroalkenes [60]. The best result in terms of reactivity and selectivity was achieved using dimethyl malonate, 1 mol% of organocatalyst **54** in THF at room temperature. Under optimal conditions, the Michael reaction of dimethyl malonate with β-nitrostyrene (*E*)-**1** proceeded well to give the corresponding adduct (*R*)-**38** with excellent yield and enantioselectivity (after recrystallization). The obtained adduct (*R*)-**38** was converted into (*R*)-Baclofen **8** hydrochloride according to the synthetic sequence demonstrated in Scheme 3.

**Scheme 15:**
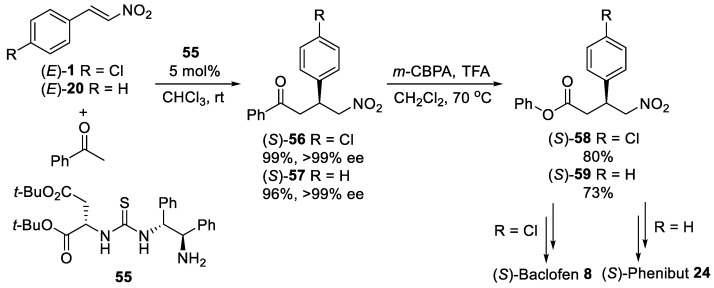
Michael reaction between acetophenone and β-nitrostyrenes using primary amine-thiourea organocatalyst **55**.

The highly stereoselective (>99% ee) conjugate addition of acetophenone to β-nitrostyrenes (*E*)-**1** and (*E*)-**20** (Scheme 15) was completed using primary amine-thiourea organocatalyst [61] based on (*S*)-di-*tert*-butyl aspartate. With the optimal organocatalyst **55**, derived from (1*R*,2*R*)-diphenylethylenediamine the Michael addition provided adducts (*S*)-**56** and (*S*)-**57** at low catalyst loading (5 mol%) in excellent yield [62]. After Bayer–Villiger oxidation of (*S*)-**56** and (*S*)-**57**, corresponding γ-nitro esters (*S*)-**58** and (*S*)-**59** could be transformed into the (*S*)-Baclofen **8** and (*S*)-Phenibut **24** according to described above procedures. The enantiomer of **55** was also utilized as organocatalyst for the efficient synthesis of (*R*)-Baclofen **8**.

**Scheme 16:**
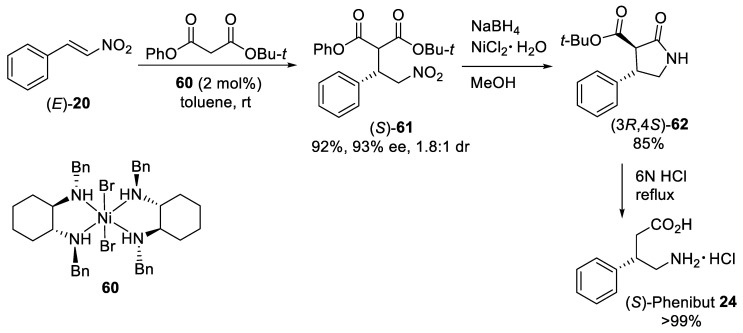
Enantioselective Michael additions of *tert*-butyl phenyl malonate to β-nitrostyrene (*E*)-**20** catalyzed by diamine−Ni(II) complex **60**.

Catalytic asymmetric version of the Michael addition reaction between malonates and nitroalkenes was also achieved by using chiral metal−ligand complexes [63,64]. For example, the highly enantioselective Michael addition of *tert*-butyl phenyl malonate to the β-nitrostyrene (*E*)-**20** (Scheme 16) was developed in toluene at room temperature using the easily accessible chiral bis-(cyclohexyldiamine)-based Ni(II) complex **60** (2 mol%) [65]. This process offered a route for the synthesis of β-nitro derivative (*S*)-**61** in 92% yield and 93% ee (1.8:1 diastereoisomeric ratio). Reduction and cyclization of (*S*)-**61** with the more reactive ester group afforded the γ-lactam (3*R*,4*S*)-**62** as one diastereomer. Final hydrolysis and decarboxylation of the γ-lactam (3*R*,4*S*)-**62** with 6N HCl under reflux produced the (*S*)-Phenibut **24** as the hydrochloride salt in quantitative yield. Later, several heterogeneous catalysts incorporating chiral bis(cyclohexyldiamine)-based Ni(II) complexes were developed for the asymmetric Michael addition of malonates to both aromatic [66,67] and aliphatic [68,69,70] nitroalkenes. These catalysts demonstrated good activities, enantioselectivities, reusability, and applicability for the multistep sequential flow synthesis of the (*S*)-Pregabalin [69].

**Scheme 17:**
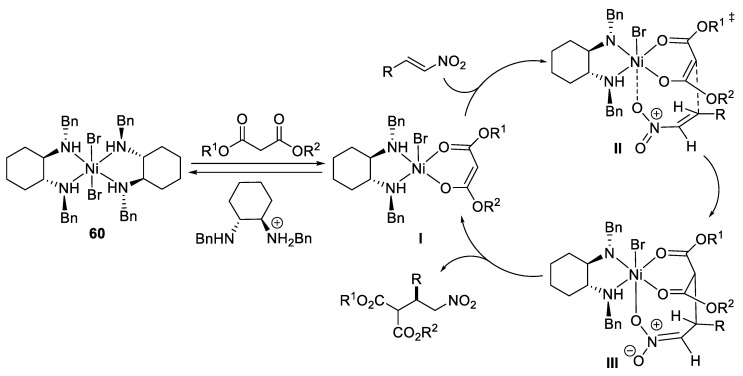
Proposed mechanism reaction.

On the basis of mechanistic studies of conjugate addition reactions catalyzed by chiral nickel(II)-diamine complexes is proposed that the malonate displaces one diamine ligand of the catalyst generating the chiral enolate **I** (Scheme 17) [65]. Coordination of the nitroalkene to the nickel center of **I** leads to the intermediate **II**, and subsequent addition of enolate to the bound nitroalkene affords the 1,4-addition intermediate **III**. Then intermolecular proton transfer and displacement of the Michael product with another molecule of the malonate regenerates chiral enolate **I**.

**Scheme 18:**
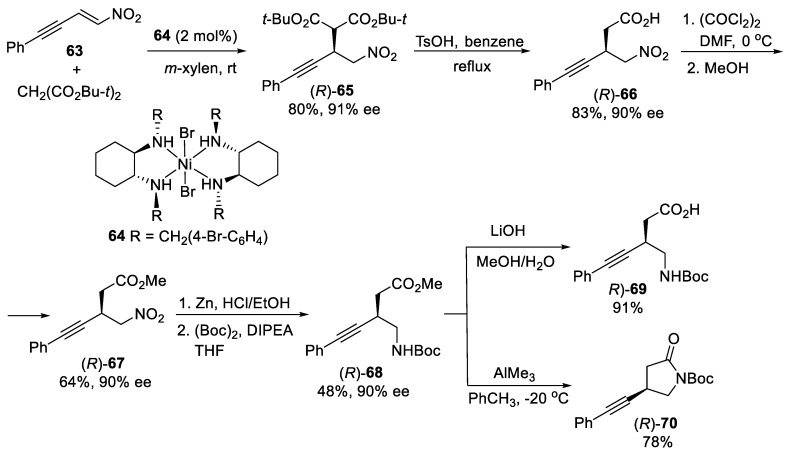
Asymmetric synthesis of β-alkynyl-γ-amino acid (*R*)-**69** and β-alkyny-γ-lactam (*R*)-**70**.

The application of the chiral nickel(II)-diamine catalysts was extended to 1,4-selective asymmetric addition of malonates to nitroenynes [71]. The 1,4-addition of di-*tert*-butyl malonate to nitroenyne **63** (Scheme 18) in the presence of 2 mol% of Ni(II) complex **64** as the catalyst proceeded regioselectively under mild conditions, affording β-alkynyl nitro acid (*R*)-**65** in good yield and high enantioselectivity (91% ee). Enantiomerical purity of product (*R*)-**65** could be improved to 99% ee by single recrystallization. This protocol allowed to obtain β-alkynyl acid (*R*)-**66** after decarboxylation of (*R*)-**65** in the presence of TsOH under reflux. Reaction of β-alkynyl acid (*R*)-**66** with oxalyl chloride and MeOH gave β-alkynyl ester (*R*)-**67** in 64% yield, which by reduction of the nitro group followed by the treatment with di-t*ert*-butyl dicarbonate (Boc)_2_O afforded the *N*-Boc β-alkynyl-γ-amino ester (*R*)-**68**. The ester (*R*)-**68**, as the common intermediate, was converted to the β-alkynyl-γ-amino acid (*R*)-**69** and β-alkyny-γ-lactam (*R*)-**70** using standard procedures without loss of enantiomeric purity.

**Scheme 19:**
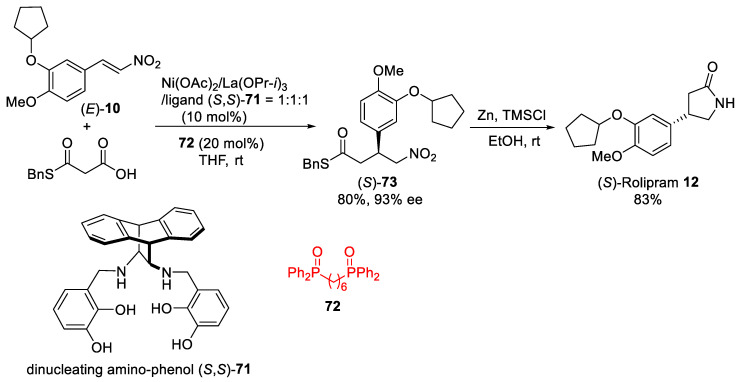
Ni–La system for decarboxylative 1,4-addition of malonic-acid half-thioester to nitroalkene (*E*)-**10**.

The catalytic asymmetric decarboxylative 1,4-addition reaction of malonic-acid half-thioester to nitroalkene (*E*)-**10** (Scheme 19) was realized by employing heterobimetallic system [72] with transition metal, rare-earth metal, and dinucleating amino-phenol ligand (*S*,*S*)-71. Screening of the transition metal and rare-earth metal combination revealed that Ni/La/ligand (*S*,*S*)-**71** catalyst in the presence of phosphine oxide **72** as an achiral additive gave the best catalytic activity and selectivity delivering the addition product (*S*)-**73** in 80% yield and 93% ee [73]. The synthetic utility of the asymmetric decarboxylative 1,4-addition reaction was demonstrated by the reduction and cyclization of (*S*)-**73** with Zn and (CH_3_)_3_SiCl to (*S*)-Rolipram **12** in 83% yield.

**Figure 2:**
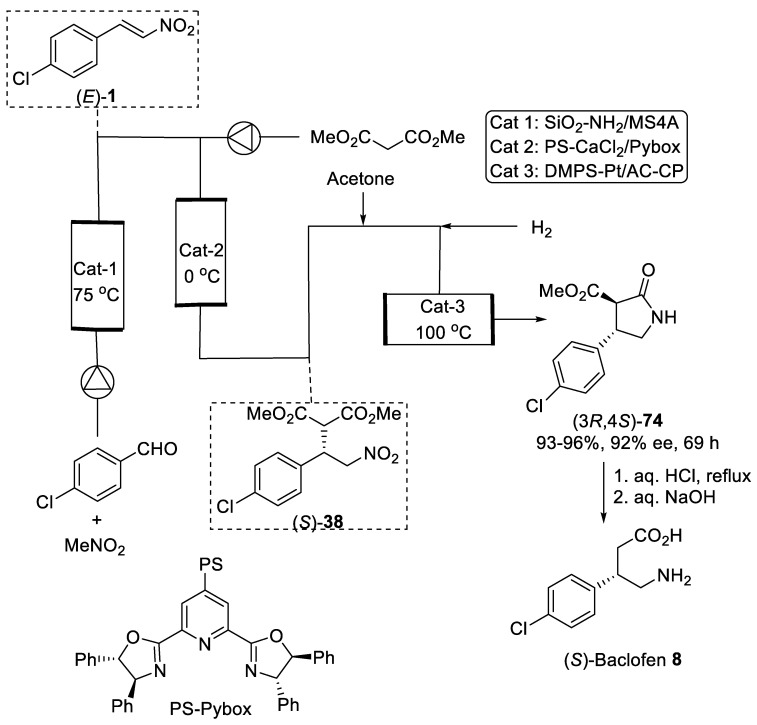
Sequential flow synthesis of Baclofen precursor (3*R*,4*S*)-**74**.

Additionally, asymmetric 1,4-addition of dimethyl malonate to β-nitrostyrene (*E*)-**1** catalyzed by polymer-supported CaCl_2_-pyridinebisoxazoline (Pybox) complex [74] was developed and implemented in continuous-flow synthesis of (*S*)-Baclofen precursor (3*R*,4*S*)-**74** using a series of different heterogeneous catalysts (Figure 2) [75]. In the first step, amine-modified silica gel/molecular sieves 4Å column reactor was used for condensation of *p*-chlorobenzaldehyde and nitromethane into β-nitrostyrene (*E*)-**1** in toluene. Then sequential-flow enantioselective asymmetric 1,4-addition of dimethyl malonate to β-nitrostyrene (*E*)-**1** proceeded into the second column reactor with polymer-supported CaCl_2_-Pybox chiral catalyst to give adduct (*S*)-**38** with high enantioselectivity (92% ee). Further, the third column reactor containing dimethylpolysilane (DMPS)-modified Pt catalyst supported on activated carbon (AC) and calcium phosphate (CP) was successfully employed for flow chemoselective hydrogenation and intramolecular cyclization of adduct (*S*)-**38** to give 5 g of lactam (3*R*,4*S*)-**73** in 93–96% overall yield based on nitromethane with 92% *ee* during the 69-h process. Finally, (*S*)-Baclofen **8** was obtained according to the standard procedure. Slightly modifying the continuous flow system using polymer-supported CaCl_2_-Pybox chiral catalyst was also employed in the synthesis of (*R*)-Phenibut [76] and (*R*)-Rolipram [77]. However, the polymer-supported CaCl_2_-Pybox chiral catalyst demonstrated poor enantioselectivities in reactions of nitroolefins bearing primary aliphatic substituents.

## 3. Michael Additions of Cyanide or Nitroalkanes to α,β-Unsaturated Carbonyl Compounds

**Scheme 20:**
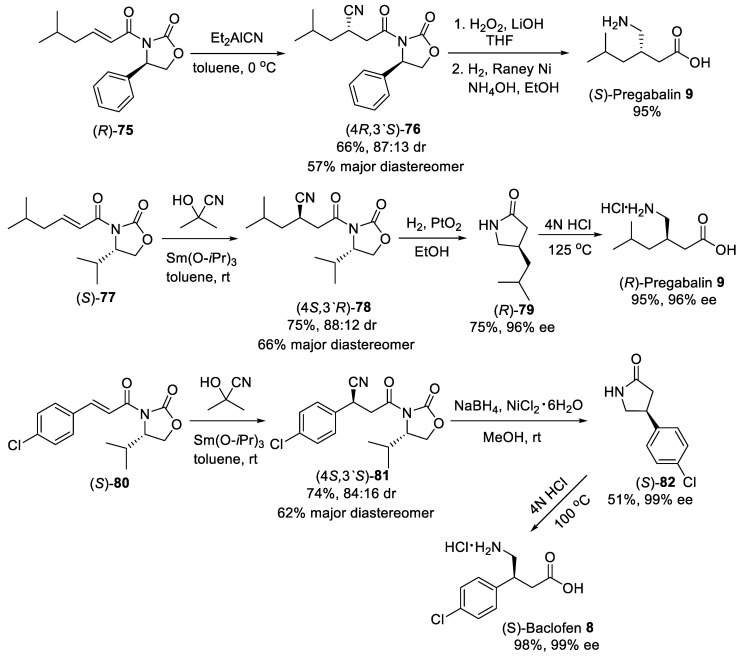
Diastereoselective Michael addition of cyanide to α,β-unsaturated oxazolidinones.

Asymmetric conjugate addition of cyanide and nitroalkanes to α,β-unsaturated carbonyl substrates was another practical route for the synthesis of β-substituted GABA derivatives. For example, the Michael addition of diethylaluminum cyanide to substrate (*R*)-**75** (Scheme 20) bearing oxazolidinone chiral auxiliary [78] was conducted as the key step in the synthesis of (*S*)-Pregabalin **9** from commercially available starting materials [79]. Conjugate addition was performed in toluene at 0 °C to produce addition adduct (4*R*,3′*S*)-**76** in satisfactory yield and moderate dr (87:13). The diastereomerically pure (4*R*,3′*S*)-**76** was provided after purification with column chromatography on silica gel in a 57% yield. The observed stereoselectivity was attributed to the approach of the diethylaluminum cyanide mainly from the less hindered *Si* face opposite to phenyl group in the oxazolidinone auxiliary. The removal of oxazolidinone chiral auxiliary by treating with LiOH and H_2_O_2_ in aqueous THF and reduction of the cyano group by hydrogenolysis under Raney Ni afforded (*S*)-pregabalin **9** in 95% yield for two steps. Moreover, acetone cyanohydrin was also effectively used as cyanide source for diastereoselective conjugate addition to α,β-unsaturated oxazolidinone (*S*)-**77** (Scheme 20) [80]. The hydrocyanation of (*S*)-**77** cleanly proceeded with two equivalents of acetone cyanohydrin and 10 mol% of Sm(III) isopropoxide as catalyst in toluene to give the addition adduct (4*S*,3′*R*)-**78** in 75% yield and 88:12 dr. The diastereomerically pure product (4*S*,3′*R*)-**78** was chromatographically isolated in 66% yield. The catalytic hydrogenation of the cyano group with simultaneous cleavage of oxazolidinone chiral auxiliary over platinum oxide afforded the lactam (*R*)-**79** in 75% yield and 96% ee, which acidic hydrolysis led to the (*R*)-Pregabalin **9** as hydrochloride in 95% yield with retention of the enantiomeric purity. A similar route was employed for the synthesis of (*S*)-Baclofen **8** using aryl-substituted substrate (*S*)-**80**, acetone cyanohydrin and Sm(O*i*-Pr)_3_ as catalyst (Scheme 20) [80]. Under standard conditions, the diastereomerically pure nitrile adduct (4*S*,3′*S*)-**81** was obtained in 62% yield. Selective reduction of the cyano group with such reagents as NaBH_4_ and NiCl_2_ and hydrolysis of resulting lactam (S)-**82** provided (*S*)-Baclofen **8** in excellent yield and enantiomeric purity.

**Scheme 21:**
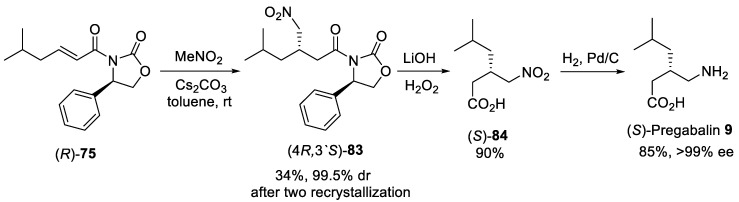
Preparation of (*S*)-Pregabalin **9**.

Similarly, oxazolidinone as a chiral auxiliary was used to generate a new stereogenic center in the Michael addition between nitromethane and α,β-unsaturated oxazolidinone ®-**75** with Cs_2_CO_3_ as a base providing a diastereomerically pure addition adduct (4*R*,3′*S*)-**83** in 34% yield after two recrystallizations from isopropanol (Scheme 21) [81]. The oxazolidinone chiral auxiliary was removed by treating (4*R*,3′*S*)-**83** with alkaline hydrogen peroxide to give the γ-nitroacid (*S*)-**84**. (*S*)-Pregabalin **9** was obtained after hydrogenation of γ-nitroacid (*S*)-**84** using Pd/C in 26% overall yield for three steps from ®-**75** with >99% ee.

**Scheme 22:**
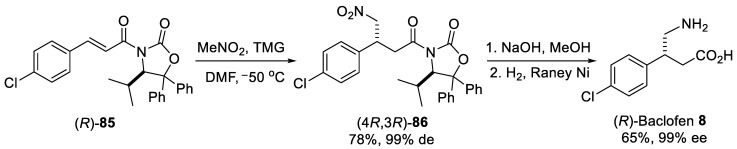
Tetramethylguanidine-catalyzed diastereoselective conjugate addition of nitromethane to α,β-unsaturated oxazolidinone (*R*)-**85**.

Chiral α,β-unsaturated oxazolidino®(*R*)-**85** (Scheme 22) was successfully used in tetramethylguanidine (TMG)-catalyzed diastereoselective conjugate addition of nitromethane with good stereoselectivity (93:7) [82]. The addition product (4*R*,3′*R*)-**86**, after crystallization from ethyl acetate, was obtained in 78% yield and 99% de. Subsequent synthetic operations involving elimination of the chiral auxiliary in basic solution, hydrogenation of NO_2_ group with Raney Ni, and recrystallization from water provided (*R*)-Baclofen **8** in 65% yield with 99% ee.

**Scheme 23:**
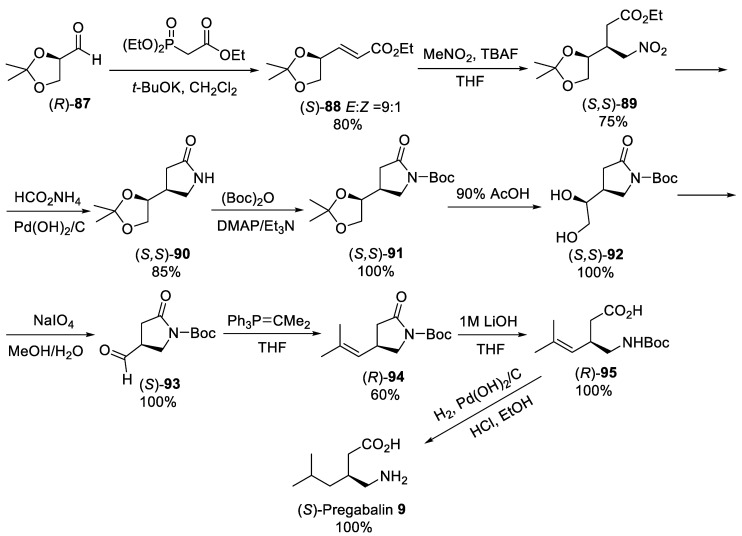
Diastereoselective synthesis of (*S*)-Pregabalin **9** from 2,3-*O*-isopropylidene-D-glyceraldehyde (*R*)-**87**.

Synthesis of (*S*)-Pregabalin **9** (Scheme 23) starting from 2,3-*O*-isopropylidene-D-glyceraldehyde (*R*)-**87** as the chiral source included highly diastereoselective Michael addition of nitromethane to α,β-unsaturated ester (*S*)-**88** that produced chiral γ-nitroester derivative (*S*,*S*)-**89** as crucial step [83]. Initially, Wadsworth–Emmons olefination of 2,3-*O*-isopropylidene-D-glyceraldehyde (*R*)-**87** with triethyl phosphonoacetate afforded α,β-unsaturated ester (*S*)-**88** as a mixture of *E*:*Z* isomers in ratio 9:1, which underwent conjugate addition of nitromethane in the presence of TBAF to give, after purification, γ-nitroester (*S*,*S*)-**89** in 75% yield. Next, the γ-nitroester (*S*,*S*)-**89** was treated with ammonium formate in the presence of Pd(OH)_2_/C, leading to the reduction of the nitro group with concomitant cyclization to the γ-lactam (*S*,*S*)-**90** in 85% yield. The corresponding *N*-Boc protected γ-lactam (*S*,*S*)-**91** was prepared by reaction with di-(*tert*-butyl) dicarbonate in the presence of DMAP and triethylamine. Then chemoselective cleavage of the ketal was achieved by using 90% aqueous acetic acid to afford diol (*S*,*S*)-**92** in quantitative yield. Oxidative cleavage of diol (*S*,*S*)-**92** with NaIO_4_ and Wittig olefination of resultant aldehyde (*S*)-**93** with isopropylidenetriphenyl phosphorane afforded β-isobutenyl γ-lactam (*R*)-**94** in 60% yield. Hydrolysis of the γ-lactam (*R*)-**94** with 1M LiOH in THF at room temperature afforded acid (*R*)-**95**, which easily hydrogenated in the presence of catalytic Pd(OH)_2_/C and *N*-deprotected by acidolysis of the Boc-carbamate with aqueous HCl to provide the enantiomerically pure (*S*)-Pregabalin **9** in quantitative yield.

**Scheme 24:**
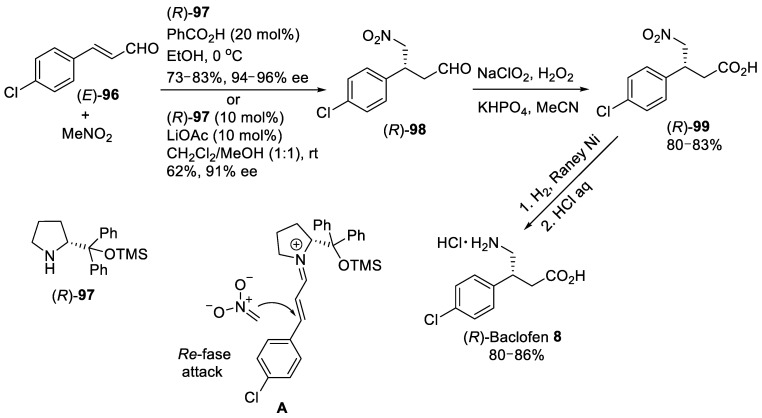
*O*-TMS-Protected prolinol (*R*)-**97** catalyzed enantioselective Michael addition of nitromethane with α,β-unsaturated aldehyde (*E*)-**96**.

The enantioselective conjugate addition of nitromethane to α,β-unsaturated aldehyde (*E*)-**96** (Scheme 24) was developed employing *O*-TMS-protected prolinol (*R*)-**97** [84,85] as organocatalyst. The asymmetric reaction catalyzed with 20 mol% of (*R*)-**97** in the presence of benzoic acid as an additive in EtOH furnished the Michael adduct (*R*)-**98** in 73–83% yield with excellent 94–96% ee [30,86]. When the asymmetric addition of nitromethane to α,β-unsaturated aldehyde (*E*)-**96** was carried out with 10 mol% loading of (*R*)-**97** and lithium acetate as an additive in CH_2_Cl_2_/MeOH, the Michael adduct (*R*)-**98** was obtained in 62% yield and 91% ee [87]. The mechanism of this process suggests that organocatalyst (*R*)-**97** would react with the α,β-unsaturated aldehyde (*E*)-**96** leading to the iminium ion **A** in which one of the enantiofaces is shielded by the large diphenyl(trimethylsilyloxy)methyl group. Thus, nucleophilic nitronate would attack iminium ion **A** from the *Re* face to form the observed (*R*)-configurated adduct **98**. The presence of benzoic acid, as well as lithium acetate as additives, could accelerate the generation of the iminium ion **A**. Successive synthetic transformation of the γ-nitro aldehyde (*R*)-**98** involving oxidation with NaClO_2_ in the presence of H_2_O_2_ and KHPO_4_ to γ-nitro acid (*R*)-**99** and hydrogenation in the presence of Raney Ni efficiently provided (*R*)-Baclofen **8** hydrochloride. In the subsequent study preparation of (*R*)-Baclofen **8** from commercially available materials was also accomplished by employing a one-pot synthetic procedure that involved DBU catalyzed aldol condensation of *p*-chlorobenzaldehyde and acetaldehyde, *O*-TMS-protected prolinol mediated asymmetric Michael addition of nitromethane in the presence of formic acid as an additive, oxidation, and reduction [88]. The one-pot procedure afforded (*R*)-Baclofen **8** in 31% overall yield with high enantioselectivity (93% ee).

**Scheme 25:**
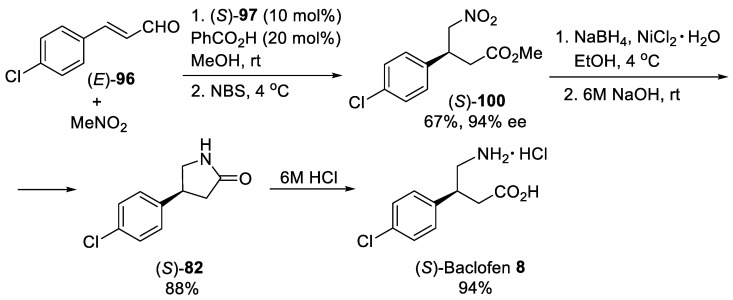
Application of the one-pot process for the formation of γ-nitroester (*S*)-**100** in the synthesis of (*S*)-Baclofen **8**.

A one-pot procedure for the synthesis of γ-nitroester (*S*)-**100** (Scheme 25) as a key intermediate of the (*S*)-Baclofen **8** was developed based on the Michael addition of nitromethane to α,β-unsaturated aldehyde (*E*)-**96** catalyzed with diphenylprolinol silyl ether (*S*)-**97** in MeOH, followed by the oxidative esterification using *N*-bromosuccinimide (NBS) as the oxidant [89]. The γ-nitroester (*S*)-**100** was obtained in 94% enantiomeric excess. Next, the γ-nitroester (*S*)-**100** was treated with NaBH_4_ in EtOH in the presence of NiCl_2_, leading to the reduction of the nitro group with accompanying cyclization to the γ-lactam (*S*)-**82,** which upon hydrolysis with 6M HCl afforded the (*S*)-Baclofen **8** as the hydrochloride in 94% yield.

**Scheme 26:**
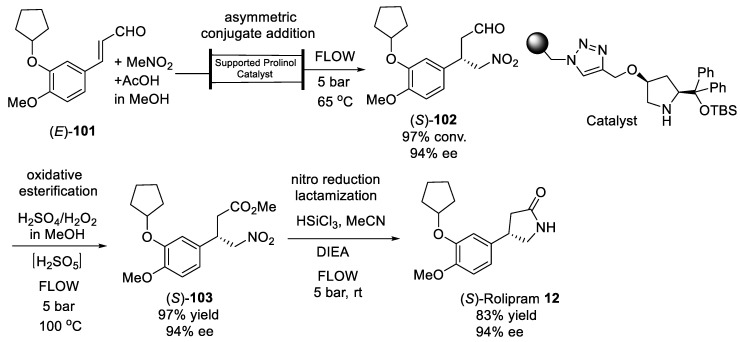
Continuous-flow enantioselective synthesis of (*S*)-Rolipram **12**.

Recently, a highly efficient process for continuous flow asymmetric synthesis [90,91,92] of Rolipram was developed. This three-step process implied (1) an initial organocatalytic flow synthesis based on telescoped flow asymmetric conjugate addition, (2) oxidative aldehyde esterification sequence using in situ-generated persulfuric acid [H_2_SO_5_] as oxidant, and (3) a final nitro reduction and lactamization. The process is initiated by an enantioselective Michael-type addition of nitromethane with the appropriately substituted cinnamaldehyde derivative (*E*)-**101** (Scheme 26) in the presence of cross-linked polystyrene-supported *cis*-4-hydroxydiphenylprolinol *tert*-butyldimethylsilyl (TBS) ether as the chiral catalyst for the formation of the γ-nitroaldehyde (*S*)-**102**, which was obtained after adjusting the reaction conditions in 94% ee and 97% of conversion [93]. Taken into account that the initial chiral γ-nitroaldehyde (*S*)-**102** was labile and could decompose during purification, it was oxidized efficiently towards the corresponding γ-nitroester (*S*)-**103** by the action of the oxidizing agent persulfuric acid [H_2_SO_5_], in situ generated by the reaction of concentrated sulfuric acid with H_2_O_2_ in a continuous-flow oxidative esterification. To complete the flow synthesis of Rolipram, a nitro-reduction was necessary, which was carried out by treatment of the γ-nitroester (*S*)-**103** with trichlorosilane (HSiCl_3_) in dry CH_3_CN. It was also observed that 4 equiv of *N*,*N*-diisopropylethylamine (DIEA) and 4 equiv of trichlorosilane were necessary to achieve quantitative and selective final nitro reduction/lactamization. In this manner, the enantioselective flow synthesis of (*S*)-Rolipram **12** was carried out after 4 h run in 83% yield and 94% ee. A similar continuous-flow process was developed for the asymmetric synthesis of substituted γ-nitrobutyric acids as key intermediates in the synthesis of Baclofen and other GABA derivatives [94].

**Scheme 27:**
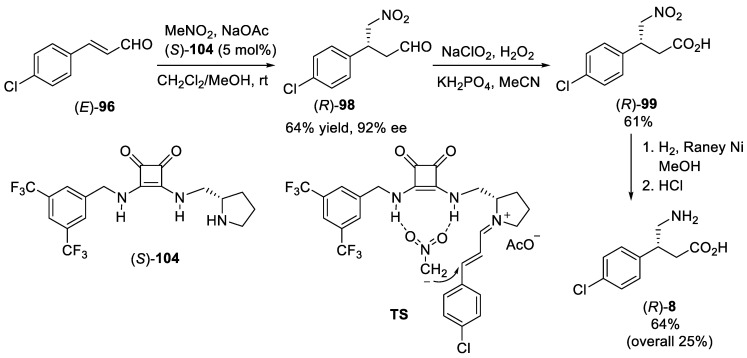
Squaramide (*S*)-**104**-catalyzed enantioselective Michael addition of nitromethane with α,β-unsaturated aldehyde (*E*)-**96**.

Bifunctional squaramide catalyst (*S*)-**104** (Scheme 27) possessing pyrrolidine moiety was employed in the enantioselective Michael additions of nitromethane to α,β-unsaturated aldehyde (*E*)-**96** [50]. The organocatalytic reaction in the best conditions provided the adducts (*R*)-**98** in synthetically valuable yield (64%) with high enantiomeric purity (92% ee). The enantioselectivity is explained through the formation of an enamine transition state **TS** allowing the *Re*-face attack of nitronate to yield the observed (*R*)-configurated adducts. Finally, compound (*R*)-**98** was converted into (*R*)-Baclofen **8** in oxidation/reduction/acid hydrolysis sequence.

**Scheme 28:**
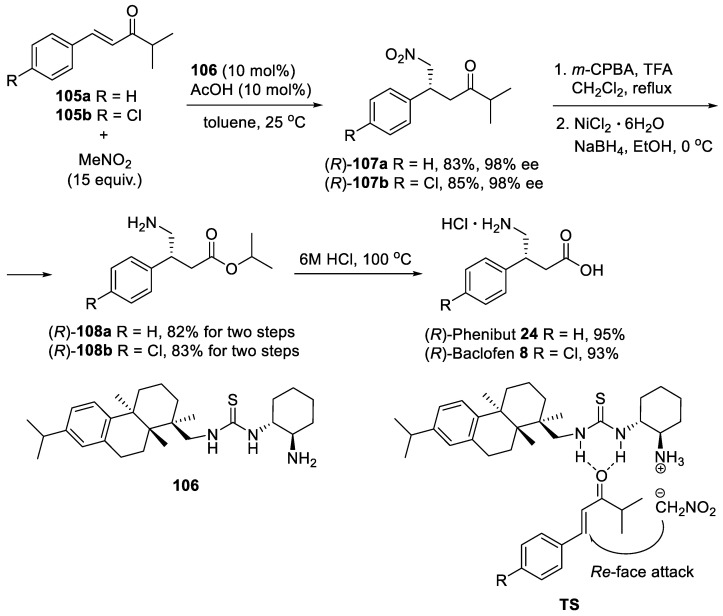
Enantioselective Michael addition of nitromethane to alkyl cinnamyl ketones and its application in syntheses of (*R*)-Phenibut **24** and (*R*)-Baclofen **8**.

Primary amine-thiourea catalyst **106** (Scheme 28) synthesized from (1*R*,2*R*)-diaminocyclohexane and dehydroabietic amine allowed one to perform asymmetric Michael addition of nitroalkanes to α,β-unsaturated ketones providing high enantioselectivities for these Michael acceptors [95]. For example, the Michael addition of nitromethane to alkyl cinnamyl ketones **105** in toluene as the optimal solvent gave the γ-nitro ketones (*R*)-**107** in 98% ee. It should be noted that excess nitromethane and the use of acetic acid as an additive shortened the reaction times and increased the yield of the desired products (*R*)-**107** to 83–85%. Compound **105** is activated via hydrogen-bonding interactions between two NH moieties of the thiourea catalyst **106** and the carbonyl group. Moreover, the amine group of the catalyst **106** provides the *Re* face approach of nitromethane in **TS** leading to formation of Michael adducts **107** with *R*-configuration. Subsequent Baeyer–Villiger oxidation then reduction of the nitro group gave the γ-amino esters (*R*)-**108** in an excellent yield. Finally, acid hydrolysis of (*R*)-**108** provided the target (*R*)-Phenibut **24** and (*R*)-Baclofen **8** in 95% and 93% yield, respectively.

**Scheme 29:**
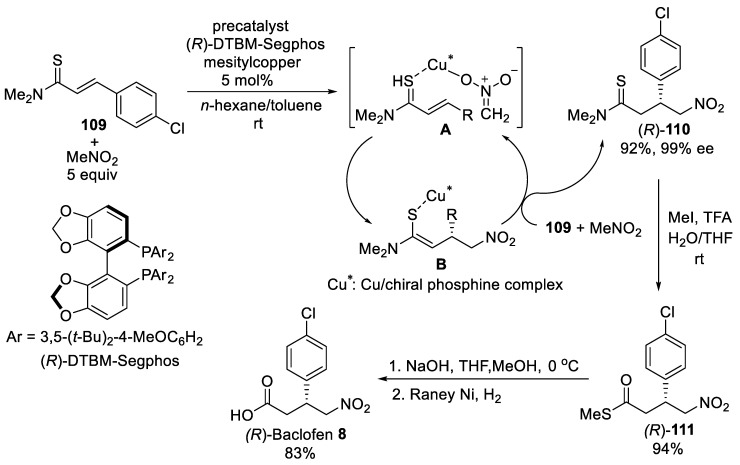
Catalytic asymmetric conjugate addition of nitromethane to α,β-unsaturated thioamide **109**.

Enantioselective conjugate addition of nitromethane to α,β-unsaturated thioamide **109** (Scheme 29) was promoted by a mesitylcopper/(*R*)-DTBM-Segphos precatalyst exhibiting dual functions to activate both pronucleophile and electrophile. The reaction was carried out at 5 mol% precatalyst loading in *n*-hexane/toluene solvent system at room temperature affording γ-nitrothioamide (*R*)-**110** in 92% yield with high enantioselectivity 99% ee [96]. Initially, deprotonation of nitromethane with chiral phosphine ligand/mesitylcopper delivering Cu-nitronate and subsequent coordination of thioamide **109** generated complex **A** (Scheme 29). Then, enantioselective C-C bond formation gave the intermediate Cu-thioamide enolate **B,** which as a soft Lewis acid/hard Brønsted base cooperative catalyst promoted proton exchange with nitromethane to give the addition adduct (*R*)-**110** with simultaneous regeneration of complex **A**. Transformation of the γ-nitrothioamide (*R*)-**110** to γ-nitrothioester (*R*)-**111** was achieved in high yield by in situ *S*-alkylation and hydrolysis with MeI/TFA in wet THF. Finally, γ-nitrothioester (*R*)-**111** was converted into (*R*)-Baclofen **8** by standard hydrolysis and reduction, highlighting the synthetic utility of the present protocol.

## 4. Michael Addition of Organometallic Reagents to α,β-Unsaturated Carbonyl Compounds

**Scheme 30:**
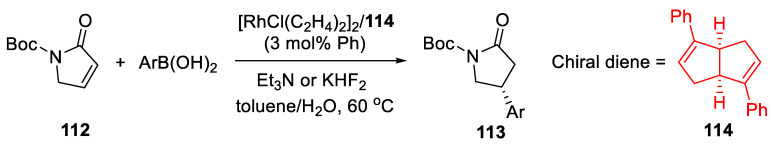
Rhodium-catalyzed asymmetric 1,4-addition reaction of arylboronic acids to α,β-unsaturated γ-lactams **112**.

Γ-Lactams are widely used as starting materials for numerous syntheses, since this backbone can be readily converted into other functional groups through many conventional organic transformations. In particular, a large number of enantioselective syntheses have been reported that use γ-unsaturated lactams as substrates for obtaining compounds, possessing a C-4 chirality, of biological or synthetic interest. In this context, the group of Lin, Feng et al. in 2011 [97,98] described an enantioselective synthesis of β-substituted γ-lactams **113** (Scheme 30) by asymmetric 1,4-addition of arylboronic acids to *N*-Boc-protected α,β-unsaturated γ-lactams **112**, a process that takes place in the presence of rhodium complexes (i.e., [RhCl(C_2_H_4_)_2_]_2_) as catalysts and using the chiral bicyclo [3.3.0]diene **114** as ligand. The designed chiral diene ligands were easily prepared by the same group in a three-step sequence [99]. An additive such as Et_3_N or KHF_2_, depending on the nature of the starting arylboronic acid, was necessary for this reaction. The process worked well, and excellent chemical yield and enantioselectivity were obtained in all the cases. However, in the case of arylboronic acids with electron-withdrawing substituents, a prolonged reaction time was required to reach the same high level of enantioselectivity and chemical yield.

**Scheme 31:**
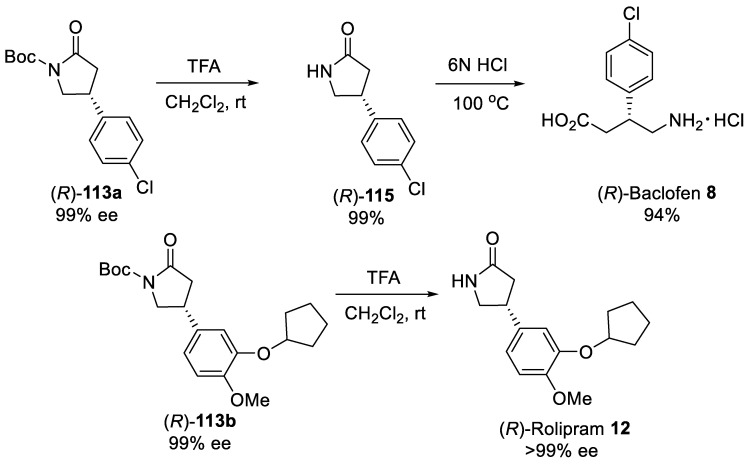
Synthesis of (*R*)-Baclofen **8** and (*R*)-Rolipram **12**.

This methodology was applied in the concise synthesis of some bioactive compounds such as the (*R*)-Baclofen **8** hydrochloride and the (*R*)-Rolipram **12** (Scheme 31). In the first synthesis, the corresponding chiral β-substituted γ-lactam (*R*)-**113a** was converted into (*R*)-Baclofen **8** in two-step by treatment with TFA, which underwent deprotection of Boc, followed by acid hydrolysis of (*R*)-**115** with 6N HCl. In the second case, (*R*)-Rolipram **12** was isolated directly after deprotection of (*R*)-**113b** with TFA in CH_2_Cl_2_ at room temperature. A high chemical yield was obtained in either case.

**Scheme 32:**
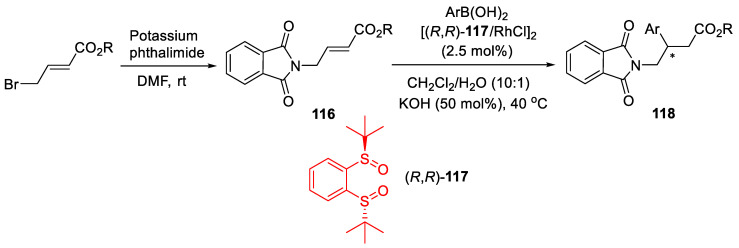
Rh-catalyzed asymmetric 1,4-addition of arylboronic acids to alkyl γ-phatalimidocrotonates **116**.

In the same year, Liao et al. [100] reported a conceptually similar procedure in a process, which implies a rhodium-catalyzed addition of arylboronic acids to γ-phthalimido-substituted α,β-unsaturated esters **116** (easily prepared from 4-bromobut-2-enoate and phthalimide potassium salt) (Scheme 32) in the presence of chiral bis-sulfoxide ligand (*R*,*R*)-**117** [101]. The process afforded β-substituted GABA derivatives **118** under mild conditions, with high yields and enantioselectivities (up to 96% ee), and using a Rh/(*R*,*R*)-1,2-bis(*tert*-butylsulfinyl)benzene complex [(*R*,*R*)-**117**/RhCl]_2_ as catalyst.

**Scheme 33:**
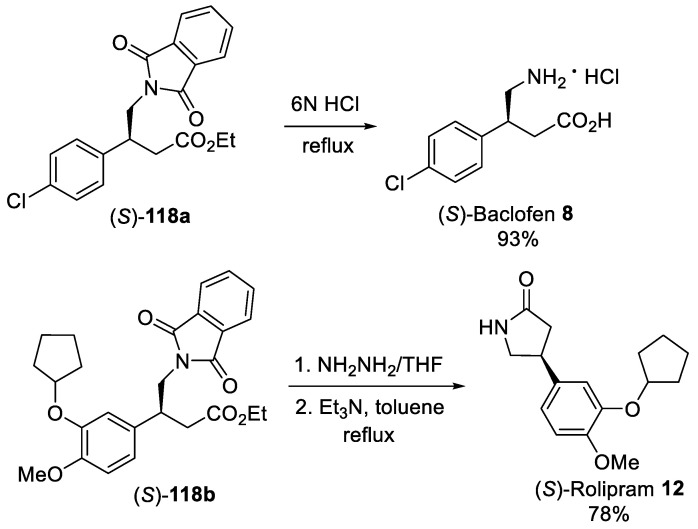
Synthesis of (*S*)-Rolipram **12** and (*S*)-Baclofen **8**.

This methodology has been successfully applied in the synthesis of the optically pure (*S*)-Baclofen **8** and (*S*)-Rolipram **12**. In the first case, a simple acidic hydrolysis (6N HCl) of (*S*)-**118a** (Scheme 33), afforded (*S*)-Baclofen **8** in 93% yield; and in the latter case, starting from the corresponding addition product (*S*)-**118b**, a two-step procedure that implies that treatment with aqueous hydrazine followed by reflux with Et_3_N in toluene provided the target (*S*)-Rolipram **12** in 78% yield.

**Scheme 34:**
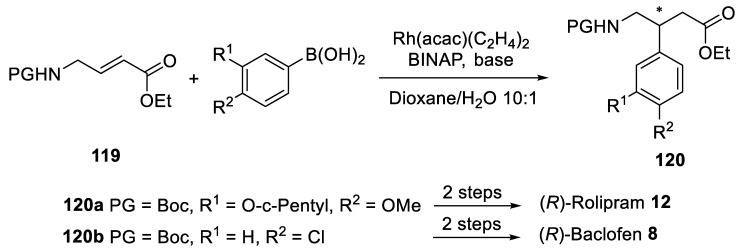
Enantioselective synthesis of (*R*)-Rolipram **12** and (*R*)-Baclofen **8** via Rh-catalyzed conjugate addition of arylboronic acids.

Another variant for the enantioselective synthesis of these two important γ-aminobutyric acid analogues was reported by Helmchen and coworkers [102], who studied the 1,4-asymmetric addition of arylboronic acids to 4-amino-2,3-enoic acid ethyl ester derivatives **119** (Scheme 34) in a process catalyzed by Rh(I) complexes and in the presence of chiral BINAP ligands. Different reaction conditions were studied, in particular the nature of the protecting groups, the base, and the ligand for preparation of adducts **120**. The best results were obtained when the base was Cs_2_CO_3_, the ligand (*S*)-BINAP, Boc as protecting group, a temperature of 100 °C, and [Rh(acac)(C_2_H_4_)_2_] as catalyst. Under these conditions and using the appropriate substituted arylboronic acid, (*R*)-Rolipram **12** and (*R*)-Baclofen **8** were obtained in good chemical yields and enantioselectivity.

**Scheme 35:**
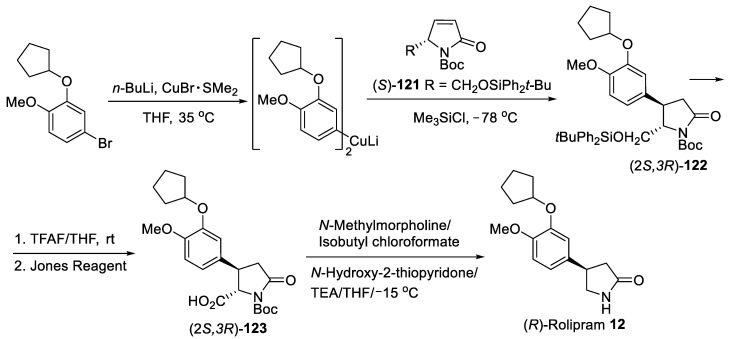
Stereoselective synthesis of (*R*)-Rolipram **12** from L-glutamic acid.

In 1997, Alvarez-Builla et al. [103] developed a quite simple and stereoselective strategy for obtaining (*R*)-Rolipram **12** (Scheme 35) starting from L-glutamic acid. The process was initiated by a conjugate Michael addition of a suitable arylcuprate to a modified pyroglutamic derivative (*S*)-**121**. The authors indicated that the steric hindrance provided by the bulky silyloxy group in the protected alcohol (*S*)-**121** (R = CH_2_OSiPh_2_*t*-Bu) was responsible for the final stereoselectivity observed. The addition product was transformed into (*R*)-Rolipram **12** by an initial deprotection of the adduct (2*S*,3*R*)-**122** with triethylammonium fluoride (TEAF), followed by oxidation of the alcohol to carboxylic acid (2*S*,3*R*)-**123** with the Jones reagent, and final simultaneous decarboxylation and deprotection of the Boc group.

**Scheme 36:**
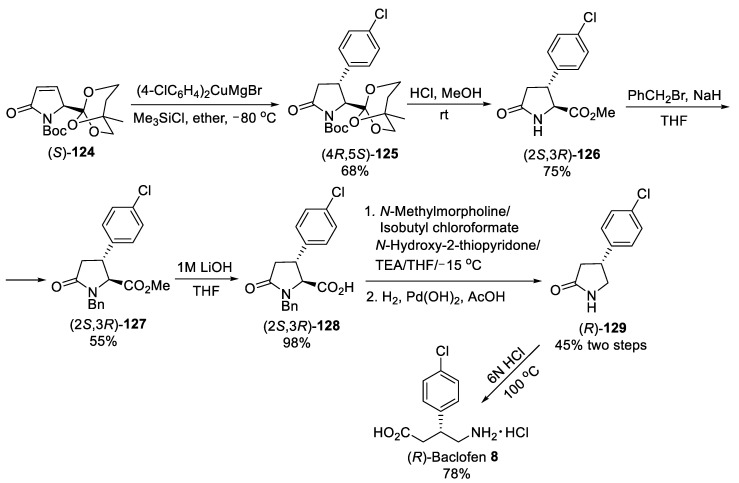
Preparation of (*R*)-Baclofen **8** using Michael addition to unsaturated pyroglutamate ester (*S*)-**124**.

The Michael reaction of unsaturated pyroglutamate derivative **124** (Scheme 36) protected as a 5-methyl-2,7,8-trioxabicyclo [3.2.1]octane ester with Grignard-cuprate containing 4-chlorophenyl group in the presence of trimethylsilyl chloride took place in good yield to give the adduct (4*R*,5*S*)-**125**. The stereoselectivity of the reaction can be explained by attacks of the organocuprate reagent at β-C of the C=C double bond from the less-hindered face [104]. The ester (4*R*,5*S*)-**125** underwent HCl-promoted methanolysis accompanying deprotection of the Boc group to give corresponding methyl ester (2*S*,3*R*)-**126** in good yield. Subsequent benzyl protection of lactam nitrogen followed by base hydrolysis of the methyl ester (2*S*,3*R*)-**127** yielded pyroglutamic acid (2*S*,3*R*)-**128**. Decarboxylation and debenzylation produced pyrrolidin-2-one (*R*)-**129,** which finally hydrolyzed by treatment with 6N HCl into (*R*)-Baclofen **8** hydrochloride [105].

**Scheme 37:**
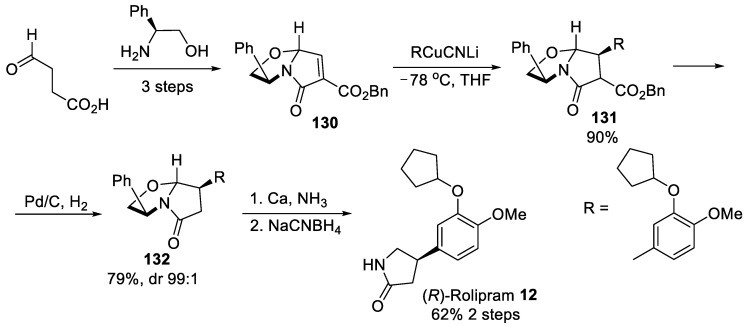
Asymmetric route to (*R*)-Rolipram **12** by addition of cuprates to chiral bicyclic lactam **130**.

An earlier route reported by Meyers [106] in 1993 allowed the asymmetric synthesis of (*R*)-Rolipram, as part of a more general study, by conjugate addition of cyanocuprates to bicycle chiral α,β-unsaturated lactams. Once the appropriate starting aryl lactam **130** (Scheme 37) had been prepared in three steps from γ-ketocarboxylic acids [107], the subsequent treatment with the appropriate cyanocuprate (−78 °C, THF) gave **131** in good yield a product derived from the conjugate addition to the exo (*β*) face of the C=C double bond. Removal of the α-carboxy function was carried out easily by hydrogenolysis followed by heating in toluene, affording **132** in good yield (79%) and excellent diastereoselectivity. The cleavage of the bicyclic system was accomplished in two steps, first by treatment with an excess of calcium metal in liquid ammonia, followed by final reduction using acidic sodium cyanoborohydride to afford the target (*R*)-Rolipram **12** in good yield. Other examples, i.e., R = Me, *n*-Bu, Ph were also reported in this relatively general study.

## 5. Miscellaneous Types of Michael Addition

**Scheme 38:**
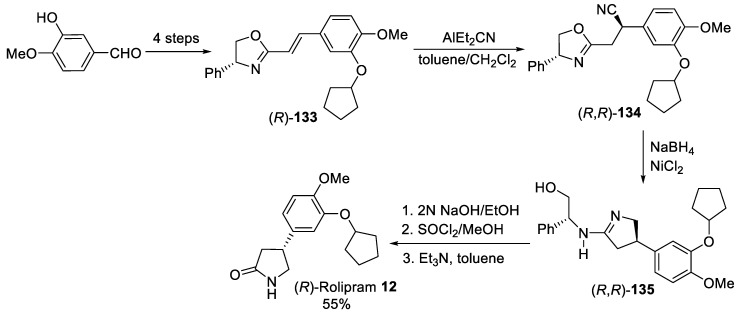
Synthesis of (*R*)-rolipram **12** using oxazolidines as chiral auxiliary.

One of the first enantioselective syntheses of (*R*)-Rolipram was developed by Langlois et al. in 1997 [108], who reported a stereoselective conjugate addition of cyanide by means of AlEt_2_CN to an activated α,β-unsaturated oxazoline (*R*)-**133** (Scheme 38), which was obtained in four steps starting from isovanillin and (*R*)-phenylglycinol as chiral auxiliary. In the process, strict control of the temperature (18 °C) was necessary to obtain an incomplete conversion (ca 50%), as at higher temperatures (i.e., 35 °C), the formation of by-products was mainly observed. Under these conditions, a mixture of two diastereoisomers, separated by flash chromatography, was obtained in 49% yield and a ratio of 63:37. The cyano group of the pure major diastereoisomer (*R*,*R*)-**134** was selectively reduced with NaBH_4_∙NiCl_2_ to the amidine (*R*,*R*)-**135**, which after alkaline hydrolysis (2N NaOH, 95% EtOH) and recovery of the chiral auxiliary was converted into (*R*)-rolipram **12** in 55% yield from the nitrile (*R*,*R*)-**134**.

**Scheme 39:**
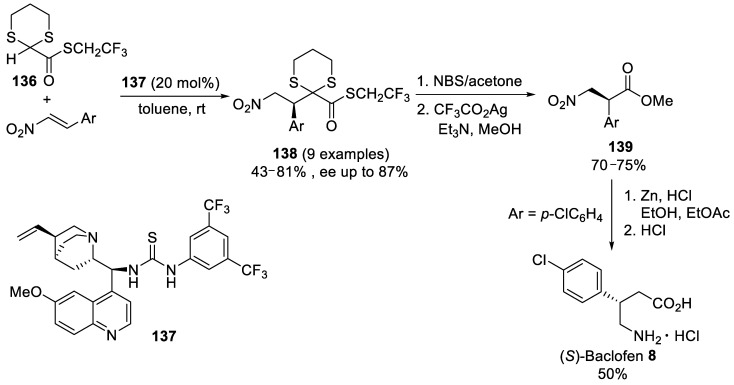
Organocatalytic 1,3-dithiane addition to styrene nitroalkenes. Synthesis of β-nitro esters and (*S*)-Baclofen **8**.

Among the different nucleophiles used for the enantioselective conjugate addition to unsaturated olefins, the α-carbanions derived from α,α-dithioacetals, which are known as *synthetic chameleons*, stand out for their relevance in the synthesis of a large number of products of biological importance. From the initial work of Koga and coworkers in 1985 [109], numerous studies have been reported emphasizing the significance of this strategy in the preparation of a large number of compounds of synthetic interest. On the other hand, electron-deficient alkenes such as nitroalkenes are important synthetic feedstocks in the construction of complex and chiral molecules in the presence of an array of stoichiometric and catalytic ligands. In this sense, in 2015 Benaglia, Gaggero et al. [110] reported the first enantioselective organocatalytic conjugate addition of 2-carboxythioesters-1,3-dithiane **136** (Scheme 39) to different nitroalkenes, particularly styrene derivatives, under mild conditions and in the presence of cinchona bifunctional derivatives as organocatalysts. Solvents such as toluene, room temperature, and cinchona derivative **137** (20 mol%) provided the best results in terms of chemical yield and enantioselectivity. Unfortunately, alkyl nitroalkenes did not react under the described reaction conditions. Conversion of the addition products **138** into β-nitro esters **139** was carried out in two steps by reaction first with NBS in acetone, followed by treatment with silver trifluoroacetate. This strategy was applied to the synthesis of the (*S*)-Baclofen **8** and other derivatives of synthetic value.

**Scheme 40:**
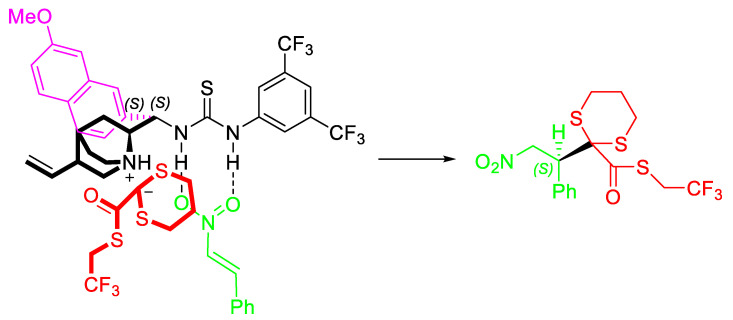
Proposed stereoselection model.

The stereochemical outcome was explained through the model depicted in Scheme 40, in which the nitroolefin is activated both by the hydrogen atom in the N-H section of the thiourea moiety and the ion pair formed between the charged quinuclidine nitrogen and the nucleophile. The thioester attack on the nitroalkene *Si* face occurs as shown in Scheme 40.

**Scheme 41:**
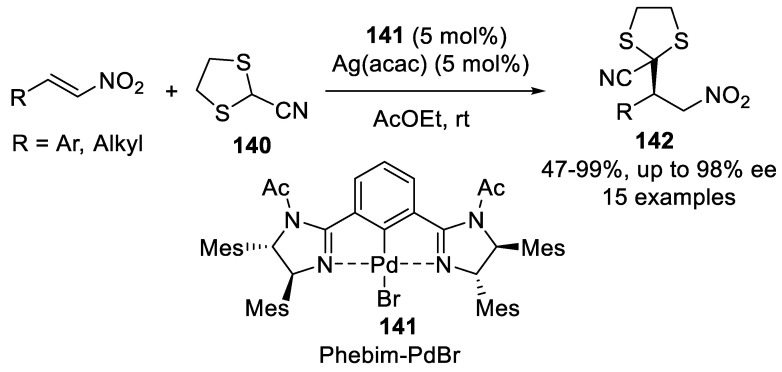
Enantioselective reaction of α,α-dithioacetonitrile **140** with nitroalkenes.

Quite recently, Nakamura et al. reported [111] a general and improved method for the catalytic enantioselective conjugate addition of α,α-dithioacetonitrile derivatives with aromatic and aliphatic β-nitroalkenes as electron-deficient alkenes, with the process being catalyzed by chiral bis(imidazoline)-palladium pincer-type complexes. In that study, the nature of the starting α,α-dithioacetal plays a crucial role in the success of the process. In addition, α,α-dithioacetonitriles are a recognized class of synthetic intermediates [112] as they can behave as cyanomethyl and cyanocarbonyl anion equivalents in their reactions with electron-deficient alkenes. After an exhaustive study with a variety of nitroolefins, 1,3-dithiane derivatives, catalysts, and reaction conditions, the authors concluded that the best results were obtained by reacting 1,3-dithiolane-2-carbonitrile **140** (Scheme 41) as α,α-dithioacetonitrile (1.5 equiv) with a variety of aromatic and aliphatic nitroalkenes in the presence of 5 mol% of the chiral palladium pincer complex Phebim-Pd complex **141** and Ag(acac) (5 mol%) at room temperature and ethyl acetate as solvent. The reaction afforded the conjugate addition products **142** with high yield and enantioselectivities (up to 98% ee).

**Scheme 42:**
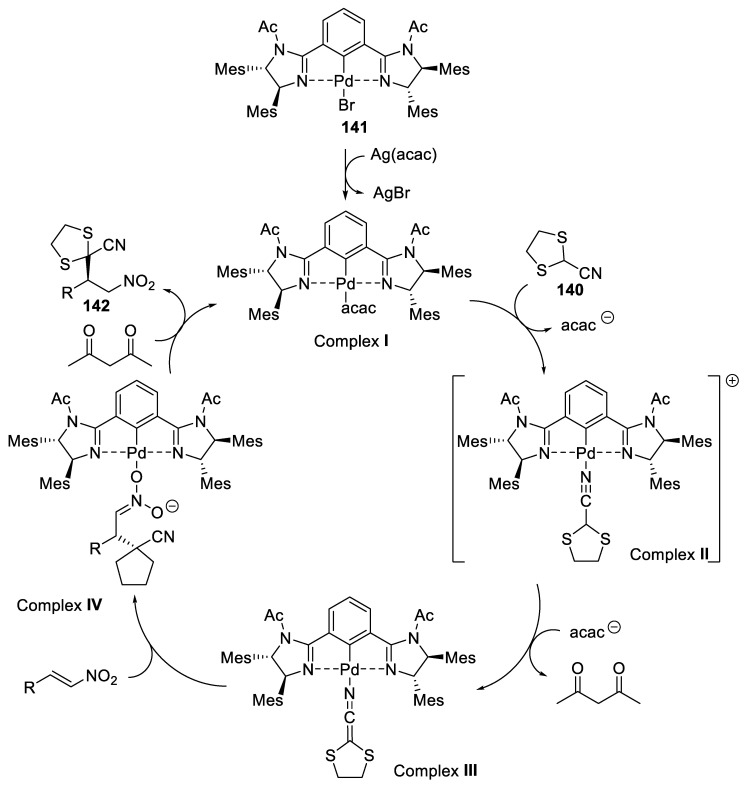
Plausible reaction mechanism for the reaction of α,α-dithioacetonitrile **140** with nitroalkenes using Phebim-PdBr **141**.

The process is quite general, being effective with electron-withdrawing, electron-rich, heteroaryl, and naphthyl aromatic nitroalkenes. Moreover, the reaction with aliphatic nitroalkenes also gave the corresponding products in good yields and enantioselectivity. It is noteworthy that many other different α-acetonitrile carbanion equivalents such as acetonitrile, phenylthioacetonitrile, cyanoacetic acid, etc., were not effective, thus indicating the essential role of the nitrile and the dithiolane groups in the global process. The proposed mechanism for this reaction is depicted in Scheme 42. The palladium acetylacetonate-complex **I** was initially formed by an exchange reaction of Ag(acac) with Phebim-PdBr **141**. This complex coordinated next with the cyano group of **140** to afford a new cationic complex **II**, which was deprotonated by acetylacetonate to give the Pd-ketenimide complex **III**. Then, the reaction of **III** with nitroalkenes took place to afford complex **IV**, which finally gave the protonated final addition product **142** and subsequent regeneration of the catalyst. This mechanism was corroborated by DFT calculations and ESI-MS spectroscopic analysis.

**Scheme 43:**
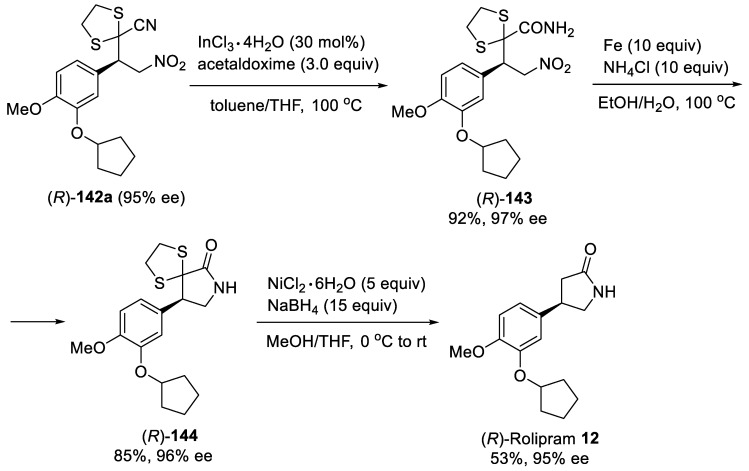
Synthesis of optically active (*R*)-Rolipram **12**.

This methodology was applied to the synthesis of γ-lactams, and particularly to the (*R*)-Rolipram **12** (Scheme 43). Thus, the previously obtained addition product (*R*)-**142a** reacted first with InCl_3_∙4H_2_O and acetaldoxime to provide the corresponding amide (*R*)-**143**, whose nitro group was reduced with Fe/NH_4_Cl. Finally, the reductive desulfurination of the γ-lactam **144** with NiCl_2_∙6H_2_O/NaBH_4_ afforded (*R*)-Rolipram **12** [113].

**Scheme 44:**
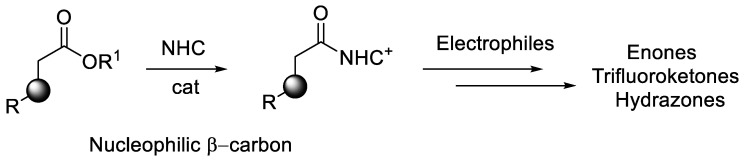
β-Carbon activation of saturated carboxylic esters.

In comparison with the enolate equivalents derived from α-carbon carbonyl compounds, the corresponding “inert” β-carbons of saturated carbonyl derivatives (β-*sp*^3^) have been far less studied in their behavior than nucleophiles [114]. In this context, Chi et al. [115] reported in 2013 an interesting β-carbon catalytic activation of simple saturated esters using *N*-heterocyclic carbenes (NHC) as organocatalysts in enantioselective reactions with various electrophiles such as enones, trifluoroketones, and hydrazones (Scheme 44).

**Scheme 45:**
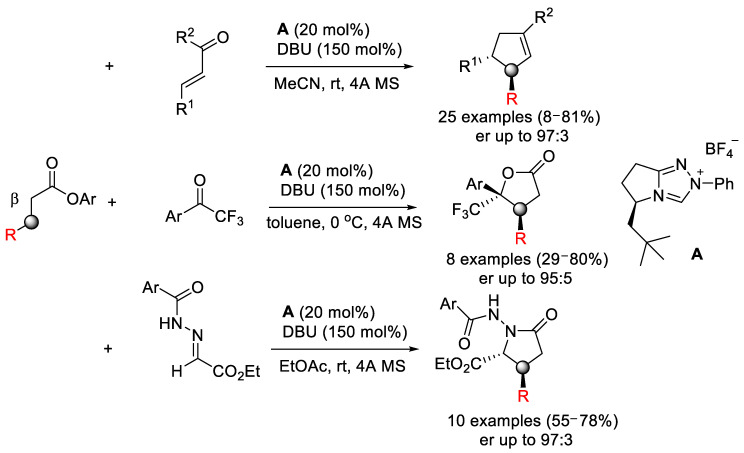
Reactivity of the ester β-carbon with different electrophiles.

After checking different reaction conditions including solvent, temperature, and achiral and chiral triazolium organocatalysts, the authors concluded that the best results in terms of efficiency (chemical yield and selectivity) were obtained by using the bulky chiral triazolium salt **A** (20 mol%) as organocatalyst in the reaction with enones as electrophiles, acetonitrile as solvent, room temperature, and in the presence of an excess of DBU as base, which was necessary to achieve the two key deprotonation steps. In this way, chiral cyclopentenes were obtained, as shown in Scheme 45. The usefulness of this strategy was also demonstrated in their reaction with other electrophiles such as trifluoroketones and hydrazones affording fluorinated γ-lactones and nonfluorinated γ-lactams, respectively. The nature of the solvent depends on the electrophile, with toluene being the solvent of choice in the case of trifluoroketones and ethyl acetate for hydrazones. The process took place, in general, with moderate-to-good chemical yields, moderate diastereoselectivity, and good enantioselectivity, obtaining the best results with β-(hetero)aryl substituted esters (Scheme 45).

**Scheme 46:**
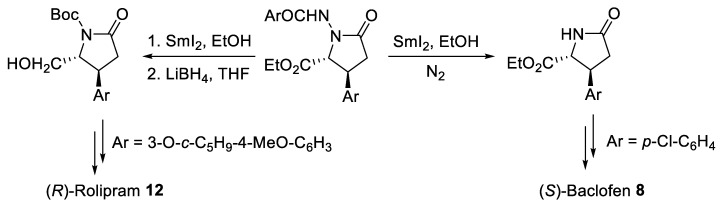
Application to the synthesis of bioactive molecules.

This methodology was used for the synthesis of bioactive molecules and building blocks of synthetic interest. Thus, chiral cyclopentenes are starting materials for optically enriched 1,2-diols and amino alcohols. γ-Butyrolactones are the key unit in the synthesis of some natural products, and γ-lactams have been used in the preparation of some pharmaceuticals such as (*S*)-Baclofen **8** and (*R*)-Rolipram **12** as shown in Scheme 46.

**Scheme 47:**
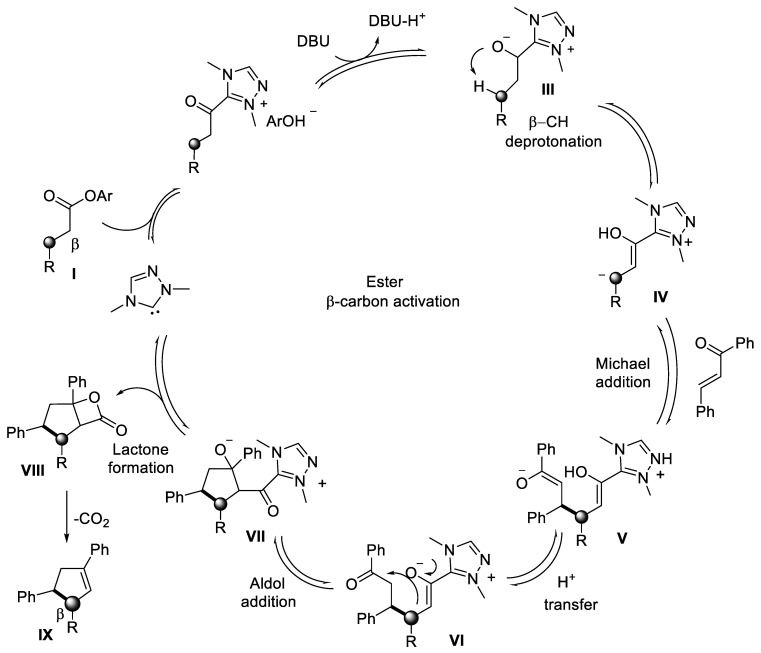
Plausible mechanism for the catalytic ester β-activation.

To explain the above-mentioned results, the authors proposed the catalytic cycle outlined in Scheme 47. The initial addition of the NHC catalyst to the ester **I** provides a new NHC-bounded ester intermediate **II**, which after deprotonation forms the enolate intermediate **III** bearing a nucleophilic α-carbon. Deprotonation of the β-CHs of enolate **III**, favored among others by the electron-withdrawing nature of the triazolium moeity, leads to intermediate **IV**. Next, the β-carbon nucleophilic center of **IV** undergoes a Michael addition with enones to give the cyclopentane intermediate **VII**, via a cascade process involving an H^+^ transfer and an aldol reaction (**V** to **VII**). Finally, lactone formation and decarboxylation provided the target cyclopentene product **IX**.

**Scheme 48:**
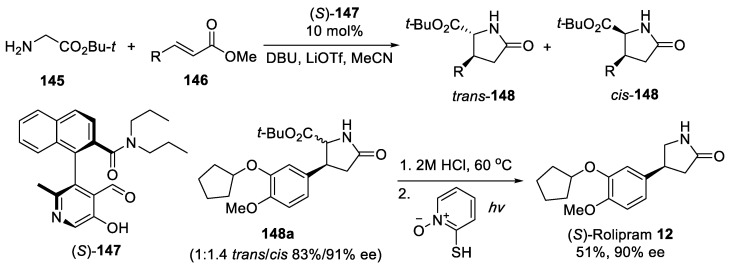
Enantioselective synthesis of pyroglutamic-acid esters from *NH*_2_-unprotected glycinate using carbonyl catalysis.

Recently, the conjugate addition of *NH*_2_-unprotected *tert*-butyl glycinate **145** (Scheme 48) to α,β-unsaturated esters **146** using chiral pyridoxal (*S*)-**147** as a carbonyl catalyst and DBU as the most appropriate base was found to produce chiral pyroglutamic-acid esters **148** after in situ lactamization with low diastereoselectivities but high enantioselectivities (81–97% ee) for both *trans*- and *cis*-diastereomers, which could be separated by column chromatography [116]. Moreover, a Lewis acid such as LiOTf was a necessary additive for the reaction. Under the optimal reaction conditions, aromatic, heteroaromatic, and alkyl α,β-unsaturated esters underwent asymmetric 1,4-conjugated addition and subsequent lactamization providing corresponding pyroglutamic-acid esters **148** in good yields with similar selectivities. Hydrolysis of the mixture of *trans*- and *cis*-diastereomers **148a** (Scheme 48) using 2M HCl and subsequent decarboxylation afforded Rolipram **12** without loss of enantioselectivity.

**Scheme 49:**
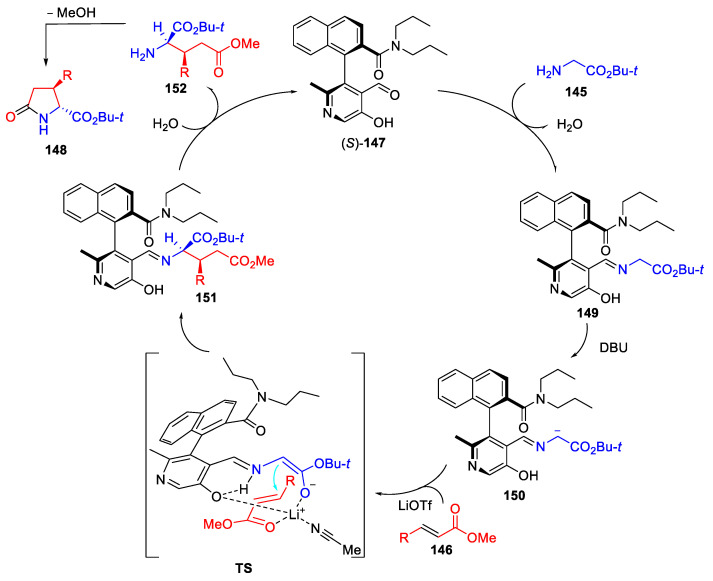
Proposed reaction mechanism.

The authors propose for conjugate addition of *NH*_2_-unprotected *tert*-butyl glycinate **145** to α,β-unsaturated esters **146** plausible pathway presented on Scheme 49. Condensation of chiral pyridoxal (*S*)-**147** with *tert*-butyl glycinate **145** gives Schiff base **149**. After deprotonation of Schiff base **149** with DBU asymmetric 1,4-conjugated addition of the carbon anion **150** to α,β-unsaturated ester **146** produces adduct **151**, which is hydrolyzed to form γ-amino ester **152** and regenerates the pyridoxal catalyst (*S*)-**147**. Finally, γ-amino ester **152** undergoes in situ intramolecular cyclization to pyroglutamic-acid esters **148**. Since *N*,*N*-propyl groups of the amide substituent in pyridoxal (*S*)-**147** catalyst shield up the side of the pyridine ring, the α,β-unsaturated ester **146** approaches the enolate anion in **TS** from direction opposite to the amide side chain, affording (2*R*,3*S*) configuration of pyroglutamic-acid esters **148** (R = Ar). At the same time, the epimerization of *trans*- and *cis*-isomers occurring under the basic reaction conditions results in observed low diastereoselectivity of reaction.

## 6. Conclusions

This review highlights the role and significance of the asymmetric Michael addition reactions as key transformations in the synthesis of β-substituted GABA derivatives used as pharmaceuticals for the treatment of central nervous system disorders. As it follows from the discussed above data, preparation of β-substituted GABA derivatives via asymmetric Michael addition reactions is a rather mature science, allowing access to this class of compounds in desired structural variety and on a large scale. In particular, considerable achievements have been made in organocatalytic conjugate addition reactions of carbon nucleophiles (malonates, aldehydes, ketones, nitroalkanes, and amino-acid derivatives) to electron-deficient alkenes as well as their synthetic applications. Numerous chiral prolinol-, (thi)ourea-, squramide-, and cinchona alkaloid-based organocatalysts developed for Michael addition reactions allow enantioselective transformations with a variety of nucleophiles under mild reaction conditions, yielding γ-nitrocarbonyl compounds with the desired stereochemistry that can be readily reduced and hydrolyzed into β-substituted GABA derivatives. Bifunctional organocatalysts bearing a hydrogen-bonding donor group are frequently employed in these types of transformations, and in many cases high catalytic activity and enantioselectivity were achieved with low catalyst loading. Metal-catalyzed asymmetric Michael reactions are also widely applied in synthesis of β-substituted GABA derivatives, providing high reactivity and stereoselectivity. In addition, organocatalysts and chiral metal complexes can be immobilized and reused without loss of catalytic activity showing a great potential for syntheses on large scale and continuous-flow multi-step preparation of β-substituted GABA derivatives. Although much progress was made in the preparation of β-substituted GABA derivatives using organo- and metal-catalyzed asymmetric Michael addition reactions, there is still opportunities for improvement of existing catalytic systems and the introduction of new catalysts with the systematic studies on the reaction mechanisms. Furthermore, the reported stereochemical data of the enantioselective reactions discussed in this review still ignore the study of the SDE-properties [117,118] of enantiomerically enriched GABA derivatives. Regretfully, SDE properties of γ-amino acids have never been reported so far. On the other hand, it has been unambiguously demonstrated that chiral amines [119,120], α- [121,122], and β-amino acids [123] persistently show a significant magnitude of SDE under a variety of transformations. As a result of the SDE ignorance, the reported values of stereochemical outcome can be recorded with sizable mistakes leading to erroneous presentation synthetic value of the corresponding reactions. Studying the SDE-properties of chiral molecules is an important issue in laboratory practice and drug development [124,125,126]. In particular, the current standards in administering chiral drugs as pure enantiomers emphasize the importance of the SDE phenomenon in the reliable and safe preparation of chiral GABA-containing pharmaceuticals [127,128].

During the revision process of this review, two interesting related reports on synthesis of α-substituted GABA derivatives [129] and synthesis of (*S*)-(+)-homo-β-proline [130] were cited, which were suggested by the referee.

## Data Availability

Not applicable.
